# Supplementary material for: An integrated toolbox to profile macrophage immunometabolism
Source: Cell Rep Methods. 2022 Mar 28;2(4):100192. doi: 10.1016/j.crmeth.2022.100192 (PMC9046227; doi:10.1016/j.crmeth.2022.100192)
Supplement: Document S1. Figures S1–S6 and Tables S1 and S2 [file mmc1.pdf]

Cell Reports Methods, Volume *2*

Supplemental information

An integrated toolbox to profile macrophage immunometabolism

Sanne G.S. Verberk, Kyra E. de Goede, Friederike S. Gorki, Xanthe A.M.H. van Dierendonck, Rafael J. Argüello, and Jan Van den Bossche

Supplementary information

Supplementary Table 1: Required optimizations for techniques, related to Table 1.

Technique	Required optimization
**Arginase assay**	Seeding density, duration of enzymatic reaction
**XF analyzer**	Seeding density, coating of plates, FCCP concentration
**SCENITH**	Timing of incubation with inhibitors and puromycin, stability of other activation or metabolic markers in flow cytometry upon treatment with inhibitors
**Metabolic dyes**	Optimal concentration and timing of dye: specificity of staining, viability, optimal fluorescent intensity; MitoTracker Green: independence of mitochondrial membrane potential

Supplementary Table 2: List of antibodies used, related to STAR Methods

Target	Species	Clone	Fluorochrome	Company	Identifier
**CD40**	M	3/23	Pacific Blue	BioLegend	Cat#124625
**CD80**	M	16-10A1	BV650	BioLegend	Cat#104732
**CD86**	M	GL-1	BV510	BioLegend	Cat#105040
**iNOS**	M	CXNFT	APC	eBioScience	Cat#17-5920-80
**CD206**	M	C068C2	BV605	BioLegend	Cat#141721
**CD301b**	M	URA-1	PE-Cy7	BioLegend	Cat#146808
**CD273**	M	TY25	PE-CF594	BioLegend	Cat#107215
**Arg1**	M	A1exF5	PE	eBioScience	Cat#17-3697-80
**F4/80**	M	BM8	BV711	BioLegend	Cat#123147
**CD11b**	M/H	M1/70	PE-Cy7	BioLegend	Cat#101216
**CD40**	H	5C3	BV785	BioLegend	Cat#334340
**CD197**	H	G043H7	BV421	BioLegend	Cat#353207
**CD80**	H	2D10	PE-Cy7	BioLegend	Cat#305218
**CD273**	H	MIH18	PE	BioLegend	Cat#345505
**CD200R**	H	OX-108	PE-CF594	BioLegend	Cat#329310
**CD206**	H	15-2	APC	BioLegend	Cat#321110
**HLA-DR**	H	G46-6	BV510	BD Biosciences	Cat#563083
**CD14**	H	63D3	AF700	BioLegend	Cat#367114
**CD68**	H	Y1/82A	PE	BioLegend	Cat#333808
**Fixable Viability Dye**	n/a	n/a	eFluor780	eBioScience	Cat#65-0865-14
**Puromycin**	M/H	R4743L-E8	AF488	SCENITH kit http://www.scenith.com/ (Arguello et al, 2020)	n/a
**MitoTracker Green**	n/a	n/a	490/516 nm	ThermoFisher	Cat#M7514
**TMRM**	n/a	n/a	548/574 nm	ThermoFisher	Cat#T668
**2NB-DG**	n/a	n/a	~465/540 nm	Invitrogen	Cat#N13195
**BODIPY C16**	n/a	n/a	505/512 nm	ThermoFisher	Cat#D3821

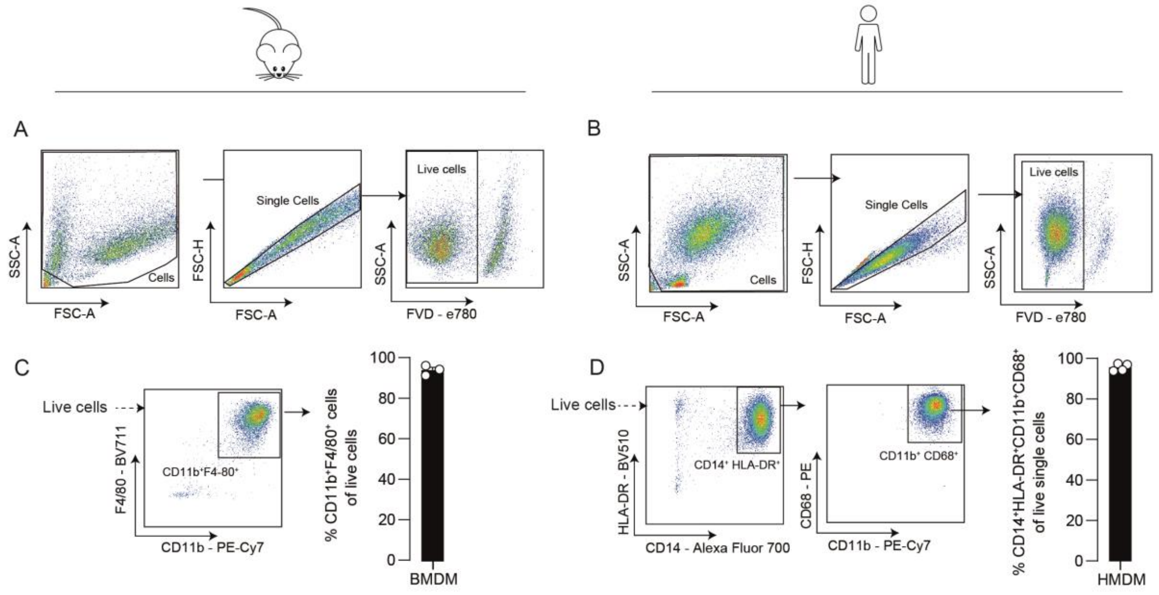

Supplementary Figure 1: Culturing of BMDMs and HMDMs leads to pure macrophage populations, related to Figures 2-5. (A, B) Gating strategy for live single cells of BMDMs (A) and HMDMs (B). (C) Gating and quantification of CD11b$^+$F4/80$^+$ BMDMs. (D) Gating and quantification of CD14$^+$HLA-DR$^+$CD11b$^+$CD68$^+$ HMDMs. Gates were set according to FMO controls. N=3 mice or N=4 donors with 4 technical replicates in 2 independent experiments.

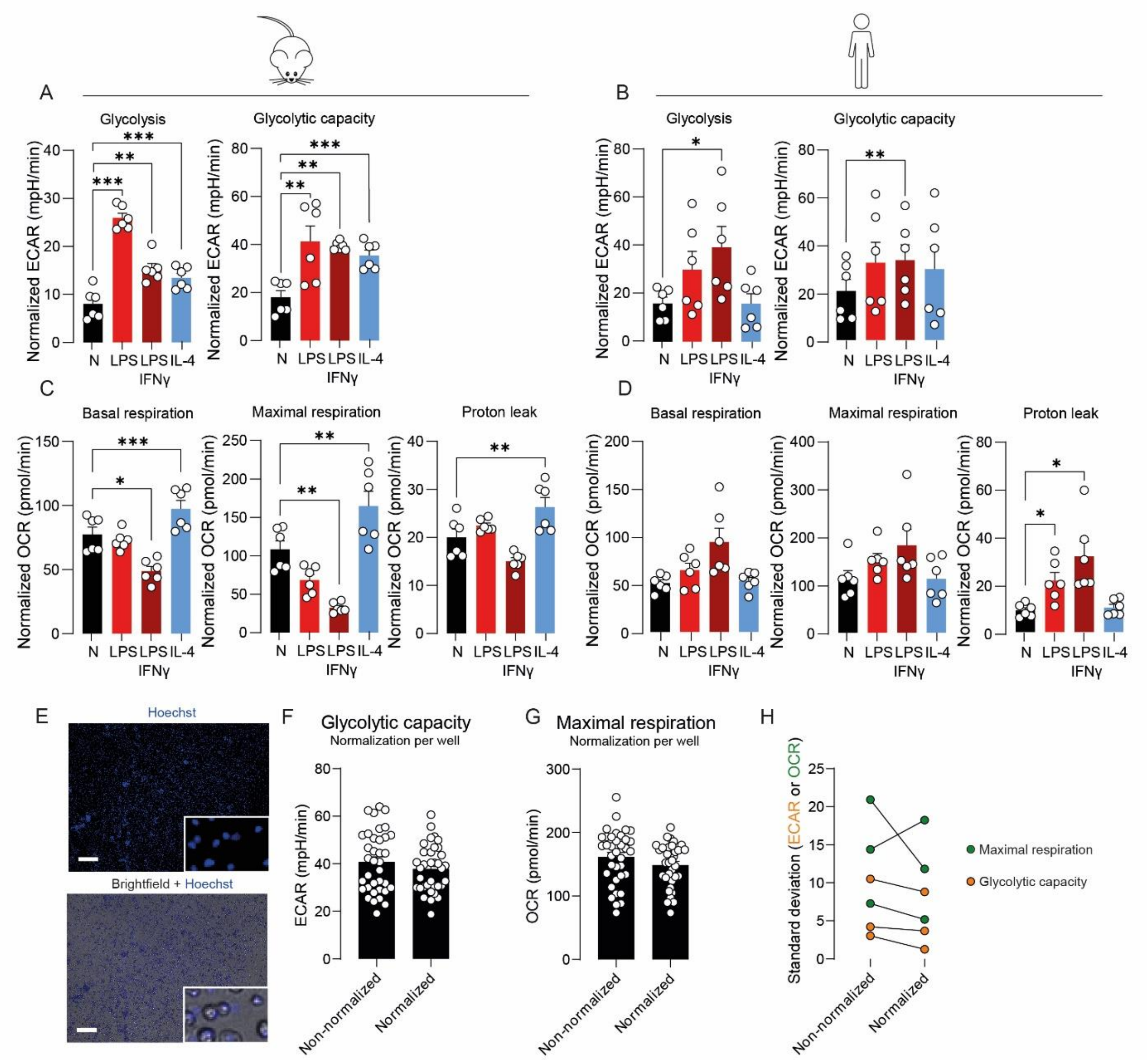

Supplementary Figure 2: Additional XF-derived parameters and normalization for cell counts after cell permeable Hoechst staining, related to Figure 3. (A, B) XF parameters extracted from ECAR data in BMDMs (A) and HMDMs (B) stimulated with LPS±IFNγ, IL-4 or left untreated. Data is normalized to relative Hoechst$^+$ objects per experiment. (C, D) XF parameters extracted from ECAR data in BMDMs (C) and HMDMs (D) stimulated with LPS±IFNγ, IL-4 or left untreated. Data is normalized to relative Hoechst$^+$ objects per experiment. (E) Hoechst staining of cells in Seahorse plate for normalization, captured in fluorescent mode and overlaid with brightfield picture to validate recognition of cells. Scale bar represents 200 µm. (F, G) Glycolytic capacity (F) and maximal respiration (G) of all measured wells before and after normalization with relative cell counts determined by Hoechst staining in 3 different experiments, each exploring unstimulated BMDMs from 3 different mice. (H) Standard deviations of XF parameters derived from combining data of 3 mice within 1 experiment, before and after normalization for relative cell count (N=3 experiments). Data are shown as mean ± SEM (A-D, F, G), individual data points indicate a mouse (N=6) (A, C), human donor (N=6) (B, D) or a separate well (F, G). Alternatively, individual data points indicate standard deviation in 1 experiment (H). * P<0.05, ** P<0.01, *** P<0.001 by one-way ANOVA with Dunnett's post-hoc test for multiple comparisons.

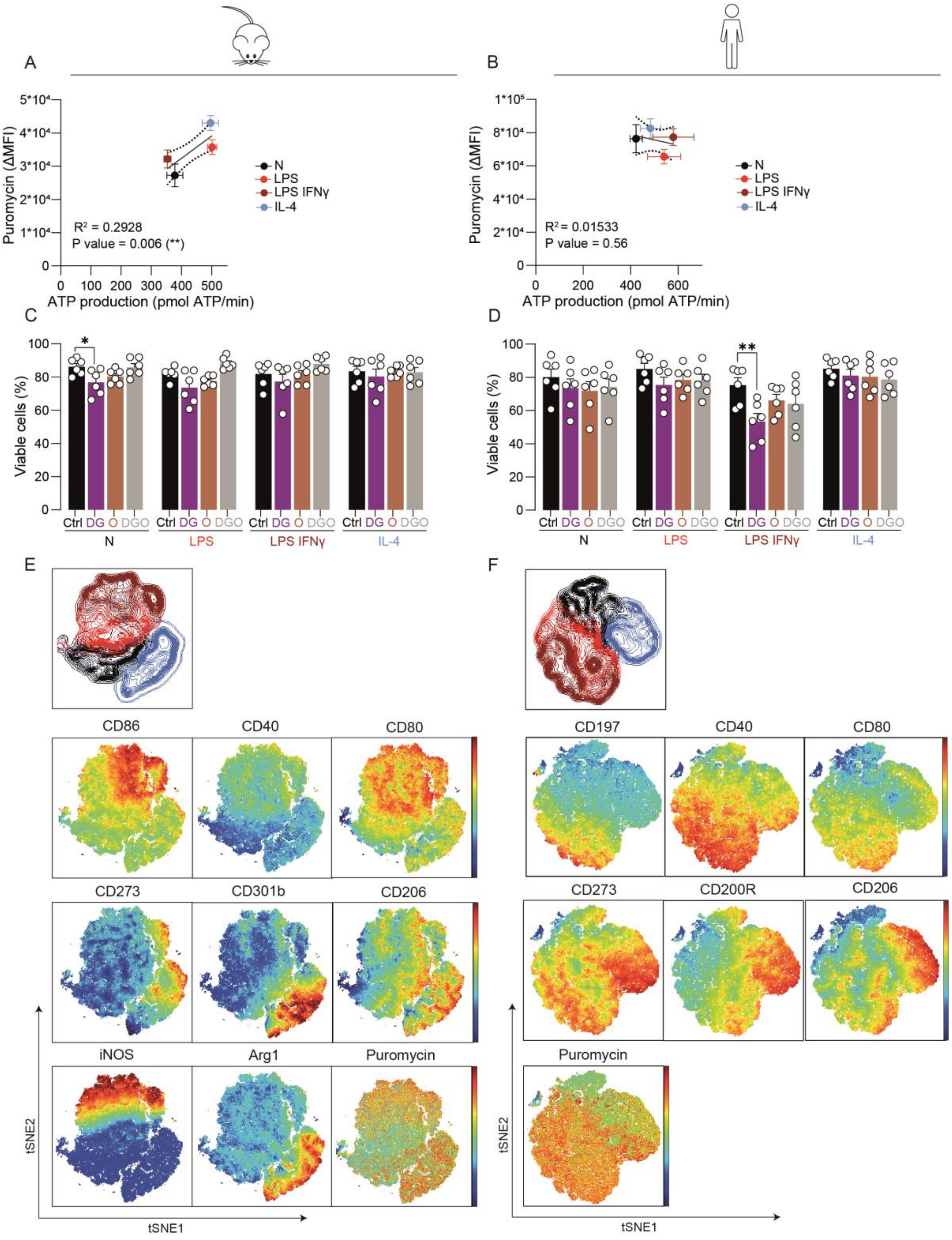

Supplementary Figure 3: Combination of SCENITH with macrophage surface markers confirms expected macrophage activation, related to Figure 4. (A, B) Correlations of ATP production as measured by XF analysis (x-axis) or by puromycin incorporation (y-axis) in mouse (A) and human (B) macrophages. (C, D) Viability of samples treated with different metabolic inhibitors during SCENITH protocol for comparison of naïve, LPS±IFNγ- and IL-4-activated BMDMs (C) and HMDMs (D) as determined by fixable viability dye. (E, F) tSNE dimensionality reduction and marker expression of oligomycin-treated BMDMs (E) and HMDMs (F). Data are shown as mean ± SEM. In C and D, each dot marks a separate mouse (N=6) or donor (N=6). * $P<0.05$, ** $P<0.01$ by two-way ANOVA with Dunnett's post-hoc test for multiple comparisons. Correlations were fitted using a simple linear regression model (A, B).

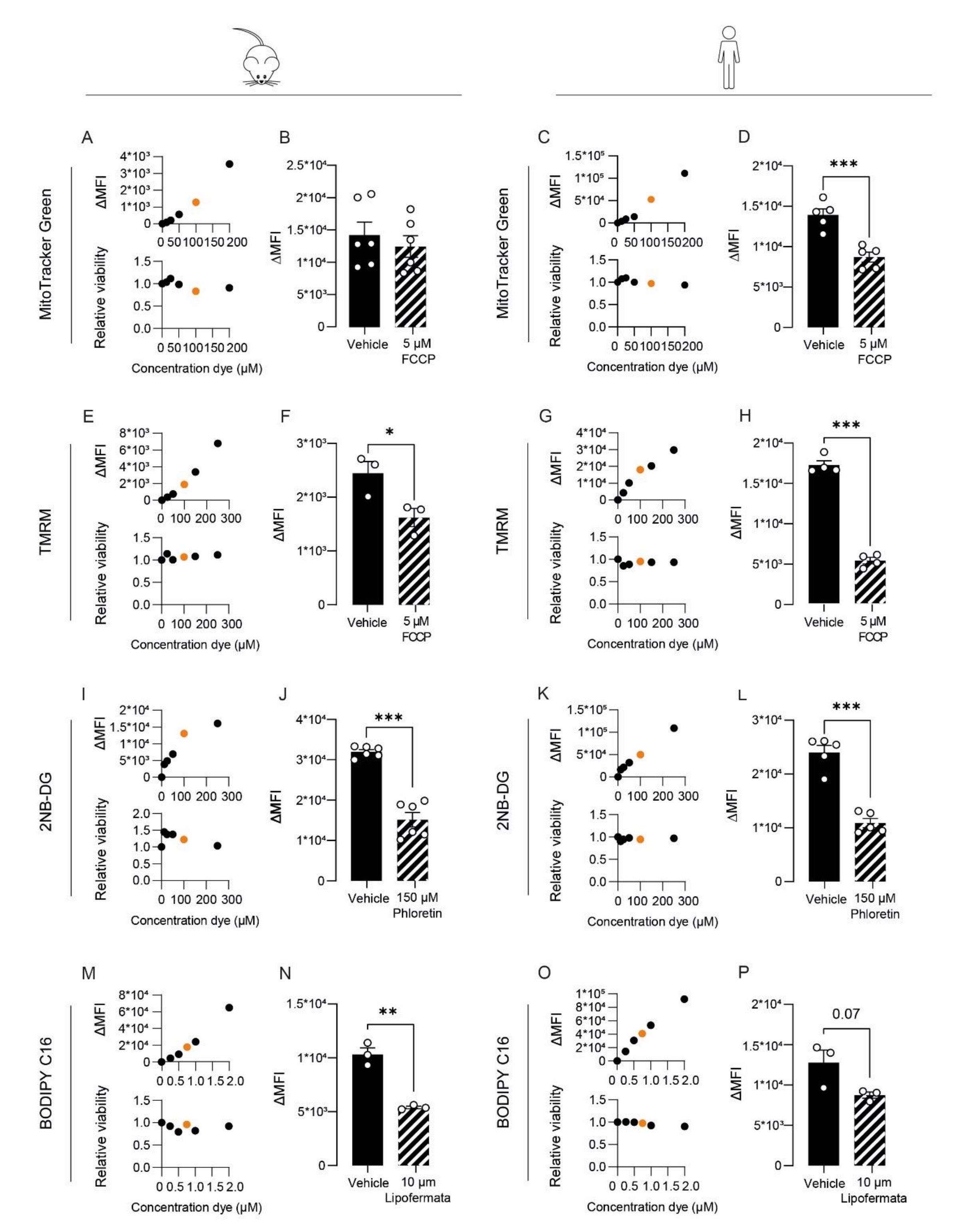

Supplementary Figure 4: Validation of metabolic dye concentration and signal measured by flow cytometry, related to Figure 5. Dose response curves of fluorescent signal and viability measured by flow cytometry or Cytation as a result of increasing metabolic dye concentration, and decrease of fluorescent signal upon inhibition for MitoTracker Green (A-D), TMRM (E-H), 2NB-DG (I-L) and BODIPY C16 (M-P) in BMDMs (left) and HMDMs (right). Orange dots indicate the concentration of the dye chosen for further experiments. Data are shown as mean from 2-3 technical replicates (A, C, E, G, I, K, M, O) or mean ± SEM where each dot marks the average of a technical duplicate for a separate mouse (N=3-6) or donor (N=3-6)(B, D, F, H, J, L, N, P). ΔMFI was calculated as MFI (median fluorescent intensity) of sample – MFI of unstained control. * P<0.05, **P<0.01, *** P<0.001 by unpaired two-tailed *t*-test.

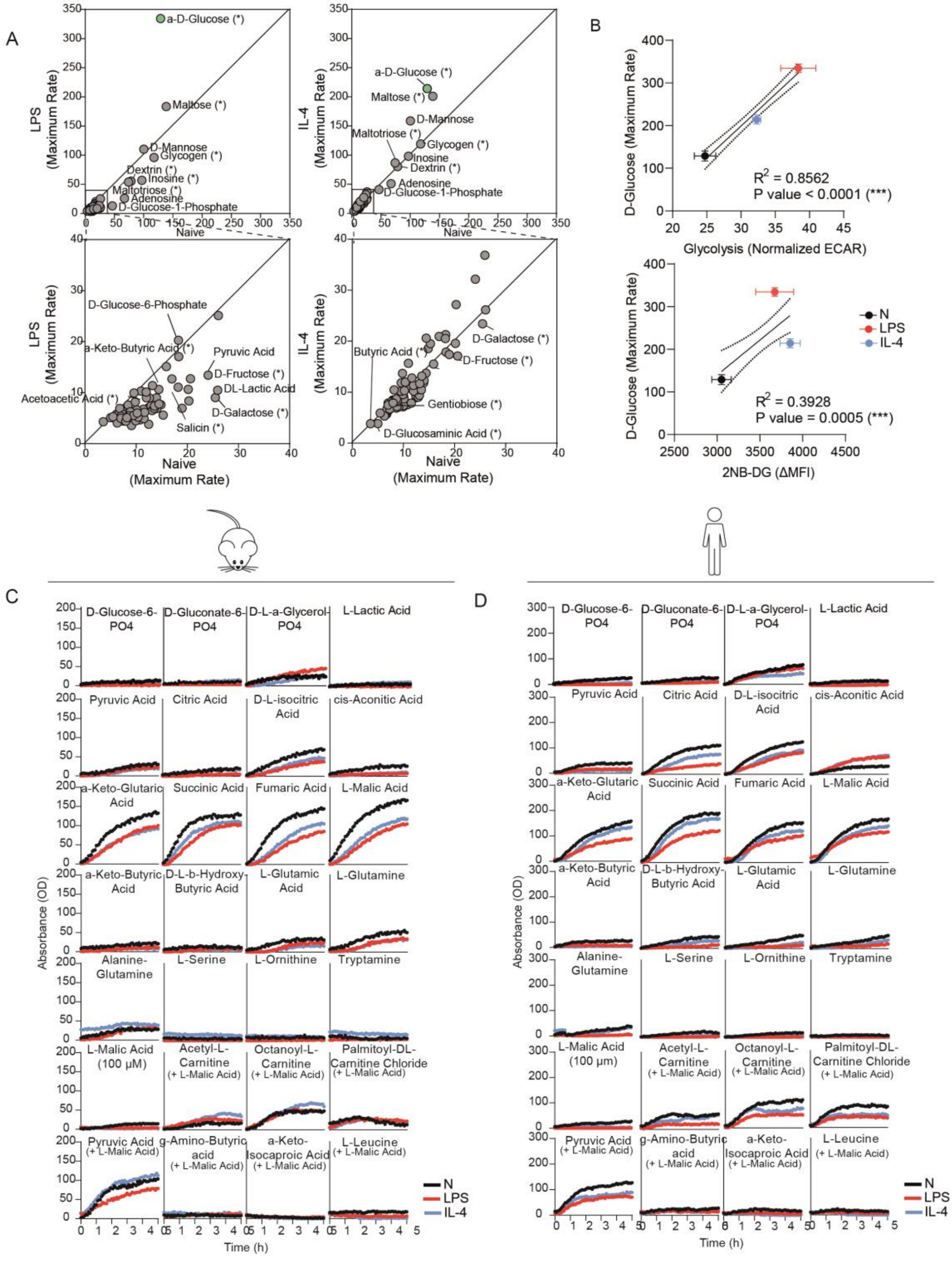

Supplementary figure 5: Assessment of substrate oxidation provides insight in glucose use and specific mitochondrial enzymatic activity by activated macrophages, related to STAR methods (Mitochondrial functional substrate assay). (A) Scatterplot comparisons of maximum rate in the timespan of 1-4h of substrate utilization by intact LPS- and IL-4-activated versus naive BMDMs. (B) Correlation of maximum D-glucose uptake as measured by carbon-substrate-coated plates and glycolysis as measured by XF analysis and 2NB-DG as measured by flow cytometry. (C,D) Kinetic rates of substrate usage by permeabilized BMDMs (C) and HMDMs (D). Correlations were fitted using a simple linear regression model (B).

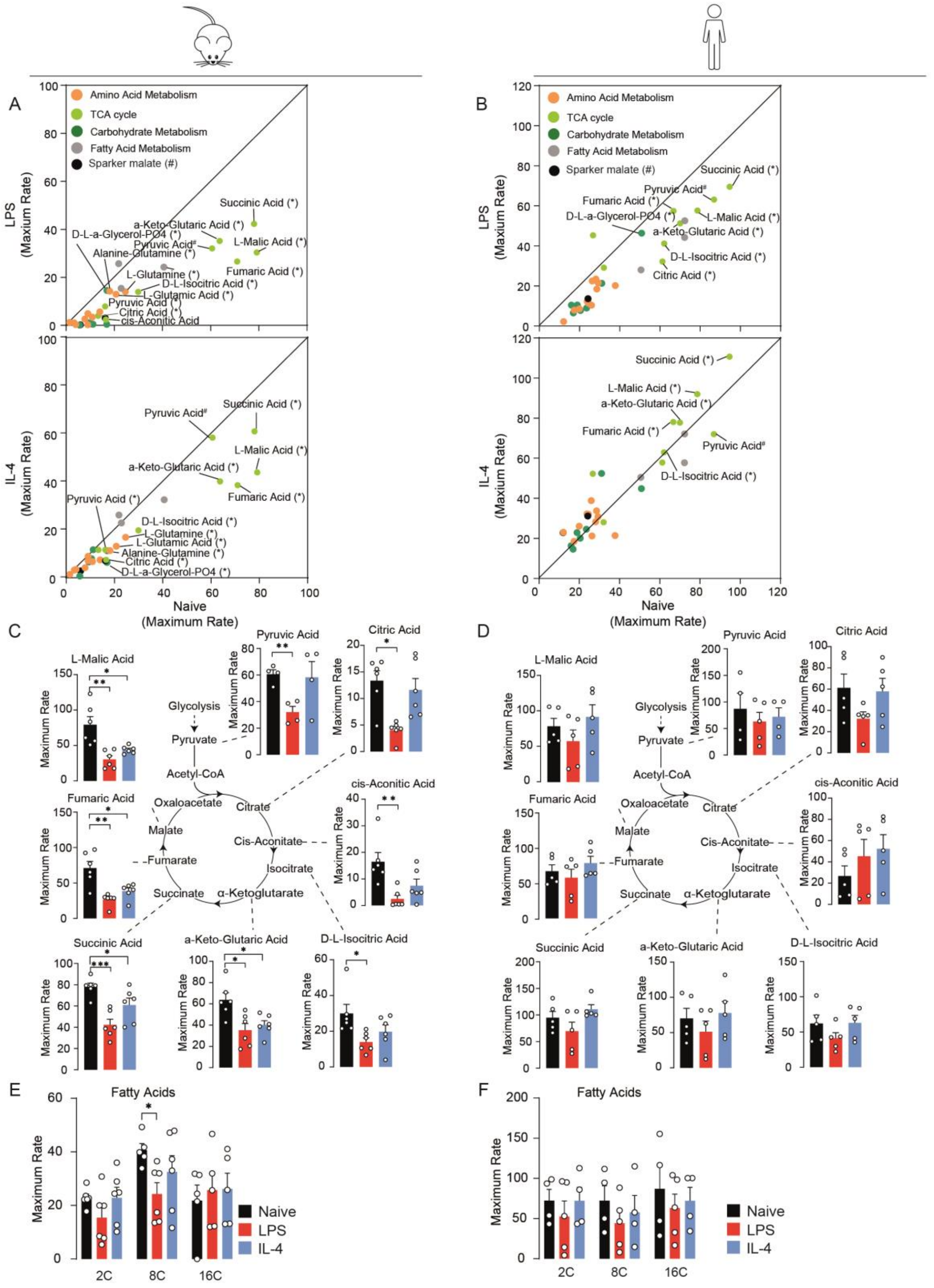

Supplementary Figure 6: Assessment of mitochondrial substrate oxidation by permeabilized cells provides insight in TCA cycle substrate and fatty acid oxidation, related to STAR methods (Mitochondrial functional substrate assay). (A, B) Scatterplot of maximum rate in the timespan of 1-4h of substrate oxidation by LPS- and IL-4-activated compared to naïve permeabilized BMDMs (A) and HMDMs (B). Substrates belonging to amino acid metabolism, TCA cycle, glycolysis and fatty acid metabolism are depicted in yellow, light green, dark green and grey, respectively. Significantly different substrates are marked with (*) and substrates supplemented with sparker malate with $^{\#}$. (C, D) Maximum rate of the oxidation of specific TCA-cycle substrates in permeabilized BMDMs (C) and HMDMs (D). (E, F) Oxidation of short- (2C), medium- (8C), and long-chain fatty acids (16C) in BMDMs (E) and HMDMs (F). Data are shown as mean (maximum) rate for all mice or donors. * $P<0.05$ by unpaired Student's t-tests (A, B).Data are shown as mean ± SEM. In C-F, each dot marks a separate mouse (N=4-5) or donor (N=4-5). * $P<0.05$, ** $P<0.01$, *** $P<0.001$ by one-way ANOVA with Sidak's post hoc-test for multiple comparisons.
