# Supplementary material for: An integrated toolbox to profile macrophage immunometabolism
Source: Cell Rep Methods. 2022 Mar 28;2(4):100192. doi: 10.1016/j.crmeth.2022.100192 (PMC9046227; doi:10.1016/j.crmeth.2022.100192)
Supplement: Document S2. Article plus supplemental information [file mmc2.pdf]

Cell Reports
Methods

Article

An integrated toolbox to profile macrophage immunometabolism

Graphical abstract

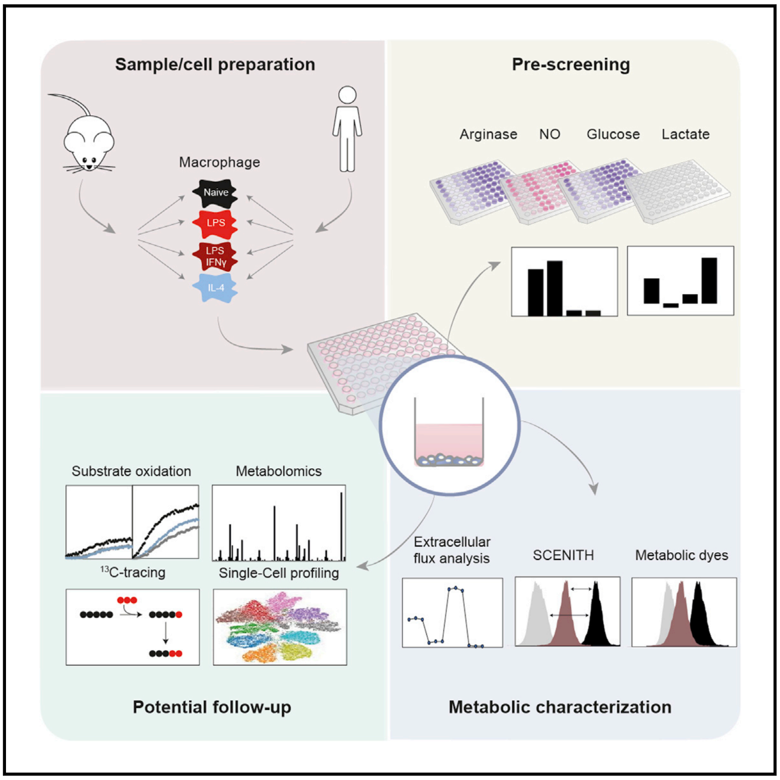

Authors

Sanne G.S. Verberk, Kyra E. de Goede, Friederike S. Gorki, Xanthe A.M.H. van Dierendonck, Rafael J. Argüello, Jan Van den Bossche

Correspondence

j.vandenbossche@amsterdamumc.nl

In brief

Verberk et al. describe accessible, stepwise workflows for profiling mouse and human macrophage immunometabolism. Their toolbox integrates distinct metabolic and functional immune assays utilizing a 96-well plate format, providing a practical, semi-high-throughput approach to assess cellular metabolism of immune cells.

Highlights

- An integrated immunometabolic profiling toolbox for macrophages
- Cost-effective approach for metabolic pre-screening prior to follow-up analyses
- Extracellular flux analysis reveals glycolysis and mitochondrial function of bulk cells
- SCENITH and fluorescent probes for *ex vivo* metabolic profiling of rare immune subsets

Verberk et al., 2022, Cell Reports Methods 2, 100192
April 25, 2022 © 2022 The Author(s).
https://doi.org/10.1016/j.crmeth.2022.100192

CellPress

Cell Reports Methods

CellPress
OPEN ACCESS

Article

An integrated toolbox to profile macrophage immunometabolism

Sanne G.S. Verberk,[1,4] Kyra E. de Goede,[1,4] Friederike S. Gorki,[1,2] Xanthe A.M.H. van Dierendonck,[1] Rafael J. Argüello,[3] and Jan Van den Bossche[1,5,*]

[1]Department of Molecular Cell Biology and Immunology, Amsterdam Cardiovascular Sciences, Amsterdam Gastroenterology Endocrinology Metabolism, Amsterdam Institute for Infection and Immunity, Cancer Centre Amsterdam, Amsterdam UMC, Vrije Universiteit Amsterdam, Amsterdam, the Netherlands

[2]Institute of Innate Immunity, University Hospital Bonn, University of Bonn, 53127 Bonn, Germany

[3]Aix Marseille Univ, CNRS, INSERM, CIML, Centre d'Immunologie de Marseille-Luminy, Marseille, France

[4]These authors contributed equally

[5]Lead contact

*Correspondence: j.vandenbossche@amsterdamumc.nl

https://doi.org/10.1016/j.crmeth.2022.100192

MOTIVATION In the last decade, it has become increasingly clear that cellular metabolism regulates immune cell function. This has sparked an expansion of immunometabolism research. However, the different techniques available may seem daunting to researchers new to the field and can also be time consuming and costly. We sought to describe and validate an integrated, 96-well-plate-based toolbox comprised of different techniques for assaying metabolic parameters in mouse and human macrophages. Next to established methods such as extracellular flux analysis and NO, arginase, lactate, and glucose measurements, the toolbox includes the recently published method SCENITH, which has not been previously applied to macrophages *in vitro*. We further discuss advantages and limitations of each technique to guide readers to effectively use the toolbox in their own research setting.

SUMMARY

Macrophages are dynamic immune cells that can adopt several activation states. Fundamental to these functional activation states is the regulation of cellular metabolic processes. Especially in mice, metabolic alterations underlying pro-inflammatory or homeostatic phenotypes have been assessed using various techniques. However, researchers new to the field may encounter ambiguity in choosing which combination of techniques is best suited to profile immunometabolism. To address this need, we have developed a toolbox to assess cellular metabolism in a semi-high-throughput 96-well-plate-based format. Application of the toolbox to activated mouse and human macrophages enables fast metabolic pre-screening and robust measurement of extracellular fluxes, mitochondrial mass and membrane potential, and glucose and lipid uptake. Moreover, we propose an application of SCENITH technology for *ex vivo* metabolic profiling. We validate established activation-induced metabolic rewiring in mouse macrophages and report new insights into human macrophage metabolism. By thoroughly discussing each technique, we hope to guide readers with practical workflows for investigating immunometabolism.

INTRODUCTION

Macrophages are innate immune cells that reside in tissues or differentiate from circulating monocytes and regulate acute inflammatory responses and tissue homeostasis (Wynn et al., 2013). While macrophages can adopt a broad spectrum of activation states, *in vitro* macrophage research primarily focuses on lipopolysaccharide (LPS)-, LPS plus interferon (IFN)γ-, and interleukin (IL)-4-induced activation states. LPS ± IFNγ-induced macrophages (classically activated macrophages, M[LPS ± IFNγ]) produce high levels of pro-inflammatory cytokines and show increased surface expression of (co)stimulatory immune activation markers. Conversely, IL-4-induced macrophages (alternatively activated macrophages, M[IL-4]) upregulate a different set of surface markers and enzymes involved in repair and homeostasis (Martinez et al., 2013; Murray et al., 2014).

Metabolic rewiring resides at the core of phenotypic polarization and has been shown to direct immune responses (Jha et al., 2015). Upon stimulation with LPS, mouse macrophages increase metabolic flux through glycolysis and the pentose phosphate

This is an open access article under the CC BY license (http://creativecommons.org/licenses/by/4.0/).

pathway (PPP), which in turn fuels reactive oxygen species (ROS) production and nitric oxide (NO) synthesis (Baardman et al., 2018; Bailey et al., 2019; Van den Bossche et al., 2017). M[LPS] mouse macrophages also display a disruption of the tricarboxylic acid (TCA) cycle at isocitrate dehydrogenase (IDH) and succinate dehydrogenase (SDH) (Jha et al., 2015). The LPS-induced downregulation of IDH results in the shunting of (iso)citrate toward synthesis of aconitate and subsequent accumulation of anti-microbial itaconate, mediated by the *immunoresponsive gene 1* (*Irg1*)-encoded enzyme ACOD1 (Lampropoulou et al., 2016). As a result of blunted SDH activity, succinate accumulates and functions as an immunoregulatory metabolite in macrophages that directs the immune response via hypoxia-inducible factor 1 alpha (HIF1α) and other mechanisms (Harber et al., 2020; Mills et al., 2016). In sharp contrast with M[LPS $\pm$ IFNγ], IL-4-stimulated mouse macrophages are characterized by an intact TCA cycle, increased fatty acid oxidation (FAO) (Van den Bossche et al., 2017; Vats et al., 2006), and increased arginase-1 (*Arg1*) activity that aid in the metabolic conversion of arginine to proline for the resolution of inflammation (Mills and O'Neill, 2016).

Although human macrophages have been less well described, they also undergo metabolic reprogramming in response to inflammatory stimuli, which slightly differs from that in mouse macrophages. First, as opposed to mouse macrophages, human macrophages do not produce NO upon stimulation with LPS $\pm$ IFNγ (Gross et al., 2014). Additionally, LPS-induced glycolysis is less pronounced or is sometimes absent in human monocytes and macrophages (Lachmandas et al., 2016; Vijayan et al., 2019). Last, the disrupted TCA cycle and mitochondrial dysfunction as seen in mouse macrophages has not yet been shown in human macrophages (Vijayan et al., 2019).

As metabolism dictates functional responses in macrophages and other immune cells, there is substantial potential in generating targeted therapeutics that combat chronic inflammatory disorders, infectious diseases, and cancer (Galli and Saleh, 2020; Lim et al., 2020; Makowski et al., 2020). In the study of new therapeutic metabolic interventions lies the importance of measuring cellular metabolism in a time- and cost-effective manner. Currently, a commonly used method is extracellular flux (XF, also known as Seahorse) analysis, which measures extracellular acidification rates (ECAR) and oxygen consumption rates (OCR) as markers for glycolysis and mitochondrial oxidative phosphorylation (OXPHOS), respectively (Van den Bossche et al., 2015). This technique has contributed to the key concept that classically activated mouse macrophages are more glycolytic, whereas mouse M[IL-4] macrophages show higher OXPHOS and FAO (Huang et al., 2014a; Tavakoli et al., 2013; Vats et al., 2006). Although the method gives a proper overview of core metabolic pathways, it does not reveal the regulation and activation of more specific cellular metabolic pathways or the direct uptake of nutrients from the environment.

A combination of several omics techniques can explain delicate changes in cellular metabolism. As such, metabolomics measures the abundance of metabolites and can reveal metabolic changes dictated by increased production or decreased substrate usage (Jha et al., 2015; Rattigan et al., 2018). Also, (single-cell) RNA sequencing (RNA-seq) (transcriptomics) shows regulation on the gene expression level and aids in evaluating the transcriptional regulation of all metabolic pathways (Artyomov and Van den Bossche, 2020; Jha et al., 2015). However, RNA-seq does not allow the assessment either of post-translational modifications that may dictate the function of metabolic enzymes or of metabolic enzyme activity. Although new methods are arising that permit the measurement of metabolic enzyme abundance and activation by (flow) cytometry (Ahl et al., 2020; Hartmann et al., 2021; Levine et al., 2021), they are relatively expensive in terms of time and costs, resulting in the inclusion of only a limited number of samples or experimental conditions. Therefore, we evaluate and integrate methods allowing for an effective screening of metabolic pathway activity to facilitate drug and inhibitor screens.

Here, we demonstrate an integrated approach in which cells and their supernatants can be used in parallel in different metabolic and phenotypic or functional readouts. While the individual methods have been published in separate papers, combining these distinct readouts facilitates a fast and cost-effective way to profile macrophage immunometabolism in a 96-well-plate-based format. First, we perform NO production and arginase activity measurements to quickly show metabolic alterations as a preliminary readout tool for mouse macrophages. Measurements of glucose consumption and lactate production serve as a first indication of altered glycolytic flux in both mouse and human macrophages. Next, we compare XF analysis with the recently developed method "single-cell energetic metabolism by profiling translation inhibition" (SCENITH) for measuring metabolism in cell subsets (Arguello et al., 2020). Additionally, we apply fluorescent dyes to assess glucose and fatty acid uptake, mitochondrial mass, and mitochondrial membrane potential. Finally, we provide a comprehensive protocol to measure specific substrate usage in intact and permeabilized cells as an optional follow-up method. The methods are applied to LPS $\pm$ IFNγ- and IL-4-induced mouse and human macrophages, providing the tools for semi-high-throughput immunometabolic research that can be applied in macrophages and may be extended to different immune cells.

RESULTS

Integration of 96-well-plate metabolic readouts into an immunometabolic profiling toolbox

We evaluated and combined existing techniques and integrated them in a toolbox to interrogate immunometabolism in a semi-high-throughput manner using 96-well plates both in parallel and consecutively.

The approach presented here includes an easy and quick pre-screening for metabolic alterations (Figure 1) that can be combined with phenotypic/functional analyses (ELISA, flow cytometry, viability, etc.) and followed up by more dedicated readouts including XF analysis and SCENITH-based analysis of cell subsets (Arguello et al., 2020) and by measuring uptake of fluorescent probes by flow cytometry or fluorescent imaging. Together, this integrated approach allows for fast (1 assay day after 1-week culture) screening of macrophage metabolism and function.

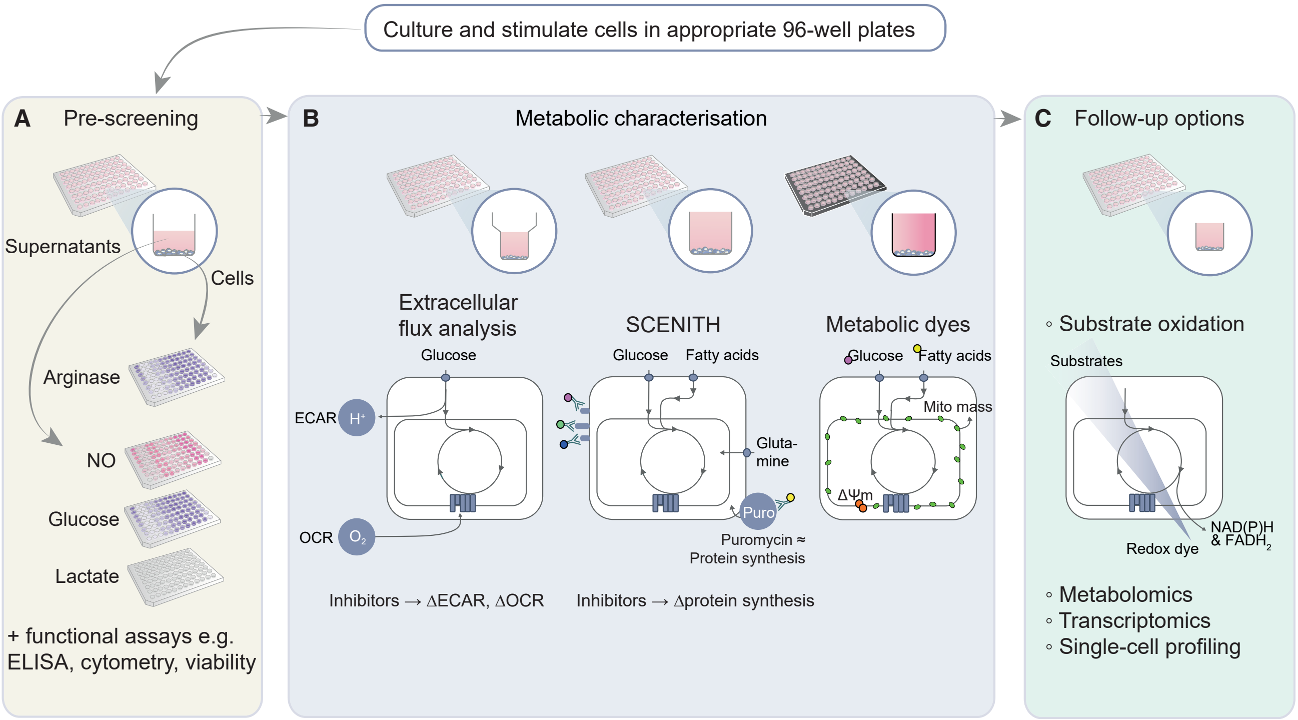

Figure 1. Overview of semi-high-throughput techniques encompassed in metabolic pre-screening and toolbox

(A) Metabolic pre-screening, consisting of arginase activity assay in cell lysates and NO, lactate, and glucose levels in cellular supernatant (yellow).

(B and C) The core metabolic characterization, consisting of extracellular flux (XF) analysis, SCENITH, and uptake of fluorescent metabolic dyes (purple). XF analysis measures extracellular acidification and oxygen consumption in XF 96-well plates in response to metabolic inhibitors to estimate glycolysis and OXPHOS, respectively. The flow cytometry-based metabolic profiling technique SCENITH (Arguello et al., 2020) measures changes in the level of translation in response to inhibitors as a measurement for cellular metabolism. This can be assessed in plate-reader-compatible FACS 96-well plates. Fluorescence measurement of the uptake of several metabolic dyes can be measured by an imaging multi-mode plate reader in black 96-well plates and by flow cytometry.

(C) These readouts can be followed up by more extensive metabolic profiling using substrate oxidation, metabolomics, transcriptomics, or various types of single-cell profiling.

Metabolic pre-screening gives first indications about altered macrophage metabolism

Mouse bone marrow cells and human monocytes were differentiated into macrophages (Figures S1A–S1D) and subsequently left untreated or stimulated with either LPS ± IFNγ or IL-4 (Figure 2A). Consistent with earlier studies, bone marrow-derived macrophages (BMDMs) displayed a LPS ± IFNγ-mediated increase of NO production by inducible nitric oxide synthase and increased IL-4-induced arginase activity (Figure 2B) (Van den Bossche et al., 2016). Confirming the literature, human monocyte-derived macrophages (HMDMs) did not show detectable NO production and arginase activity (Figure 2C).

Whereas NO/arginase measurements are not applicable to human macrophages, measuring extracellular glucose and lactate levels in macrophage supernatants are fast approaches to interrogate the LPS ± IFNγ-induced glycolytic switch in both mouse and human macrophages (Figures 2D and 2E). Increased glycolytic flux, as evidenced by increased glucose consumption and lactate secretion, is generally associated with increased inflammatory signaling and immune effector functions in macrophages and other immune cells (Freemerman et al., 2014; Voss et al., 2021).

XF analysis validates metabolic rewiring in LPS ± IFNγ- and IL-4-activated macrophages

More insight into alterations in glycolysis can be obtained by measuring ECAR via XF analysis. This method confirmed the increase in glycolysis in both mouse and human M[LPS ± IFNγ] and additionally revealed increased glycolysis in IL-4-stimulated BMDMs (Figures 3A, 3E, and S2A). An added value of XF analysis is that it simultaneously measures OCR as a proxy for mitochondrial function. Mouse macrophages displayed the expected upregulated mitochondrial respiration after IL-4 treatment (Figures 3C and S2C) and decreased OCR following LPS + IFNγ activation (Figures 3C and 3E) (Jha et al., 2015; Koenis et al., 2018; Van den Bossche et al., 2016). The calculated mitochondrial and glycolytic contribution to ATP production followed a similar pattern to OCR and ECAR parameters in the different conditions (Figure 3F). In HMDMs, LPS + IFNγ significantly increased glycolysis parameters, whereas none of the stimuli significantly affected OCR-derived readouts (Figures 3B, 3D, 3G, S2B, and S2D). Akin to mouse macrophages, the mitochondrial contribution to ATP production was significantly decreased by LPS ± IFNγ and increased by IL-4 (Figure 3H).

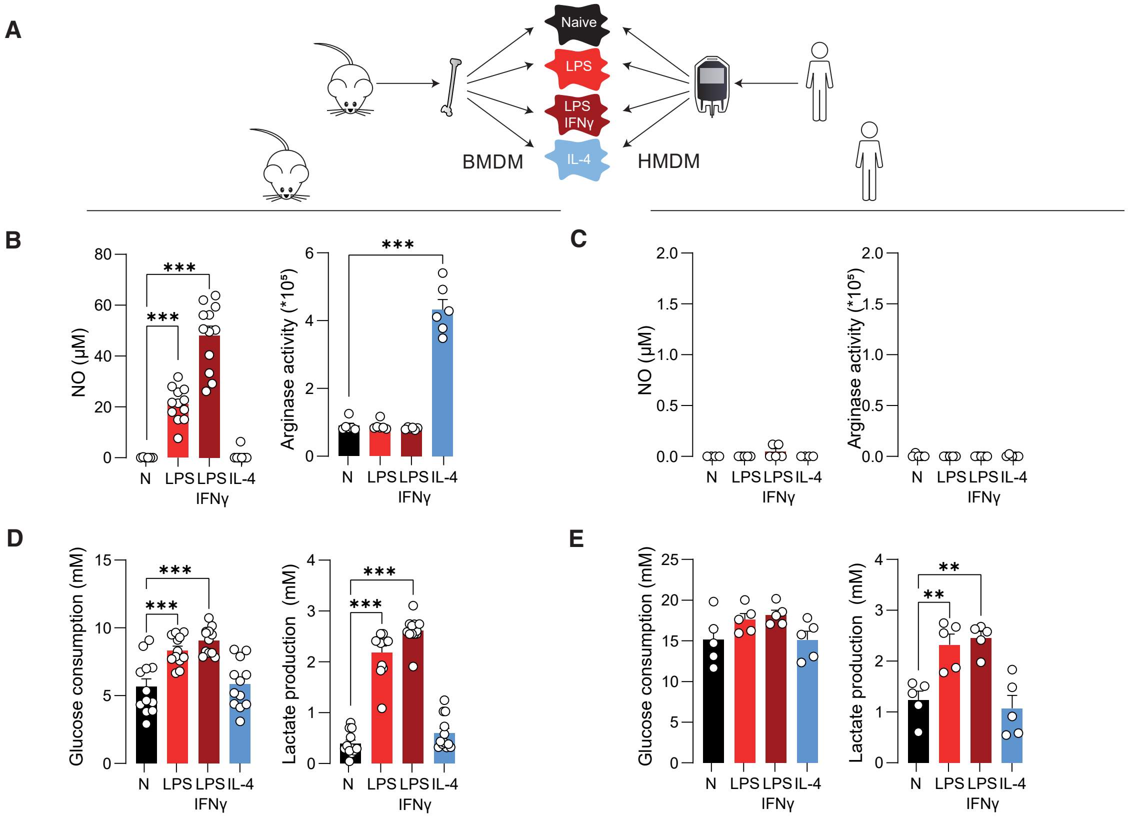

Figure 2. Metabolic pre-screening of BMDMs and HMDMs indicate metabolic differences after varying macrophage activation
(A) BMDMs and HMDMs were left untreated (naive, N), or stimulated with either LPS, LPS + IFNγ, or IL-4 for 24 h.
(B and C) Levels of NO in supernatants from BMDMs (B) or HMDMs (both 1×10^5 cells per well) (C) and arginase activity in BMDMs (5×10^4 cells per well) (B) or HMDMs (1×10^5 cells per well) (C) following stimulation.
(D and E) Levels of glucose consumption and lactate production in supernatants of BMDMs (D) or HMDMs (E) (all 1×10^5 cells per well).
Data are shown as mean ± SEM For BMDMs, n = 12 mice with three technical replicates in four independent experiments were included for NO, glucose, and lactate assays, and n = 6 with three technical replicates in two independent experiments for arginase activity assay. For HMDMs, five human donors with three technical replicates in two independent experiments were included for all assays. **$p < 0.01$, ***$p < 0.001$ by one-way ANOVA with Dunnett's post hoc test for multiple comparisons.

Since metabolic readouts like XF analysis depend on cell count, data need to be normalized. Normalization methods are further discussed in the "practical considerations" section of the discussion section. To normalize ECAR and OCR for cell counts, cell-permeable Hoechst dye was injected in the last step of XF analysis, followed by fluorescent imaging and counting as detailed previously (Little et al., 2020) (Figure S2E). Normalization of XF data with relative Hoechst$^+$ object counts (counts per well/average counts in all wells; see STAR Methods for more detail) reduced the variation between wells, as indicated by reduced standard deviations of both ECAR- and OCR-based parameters (Figures S2F–S2H).

Flow cytometry-based SCENITH method allows metabolic profiling of cell subsets and correlates well with XF analysis

Although XF analysis provides valuable insight into the glycolytic and mitochondrial function of cells, this bulk analysis requires relatively large cell numbers in the 96-well format. Moreover, it requires an XF analyzer that is not available in all laboratories. We therefore examined the SCENITH technology, which can profile the metabolism of distinct and small cell subsets (Arguello et al., 2020). This technique is based on the fact that protein synthesis and ATP levels are often tightly connected and determines the effect of metabolic inhibitors on puromycin incorporation during protein translation to estimate glycolytic and mitochondrial dependency via flow cytometry. We assessed mouse and human macrophages with SCENITH and compared it with XF analysis, as done previously for T cells (Arguello et al., 2020).

High puromycin incorporation (displayed as median fluorescence intensity, MFI) was detected in control samples, indicating a high level of protein synthesis in naive BMDMs, and this was further increased after stimulation with LPS or IL-4, which correlated significantly with XF-derived total mitochondrial plus glycolytic ATP production (Figures 4A and S3A). This was not the case for HMDMs (Figures 4B and S3B). Addition of deoxy-D-glucose (DG) for 30 min affected viability in some conditions, highlighting the need to include a viability dye (Figures S3C and S3D).

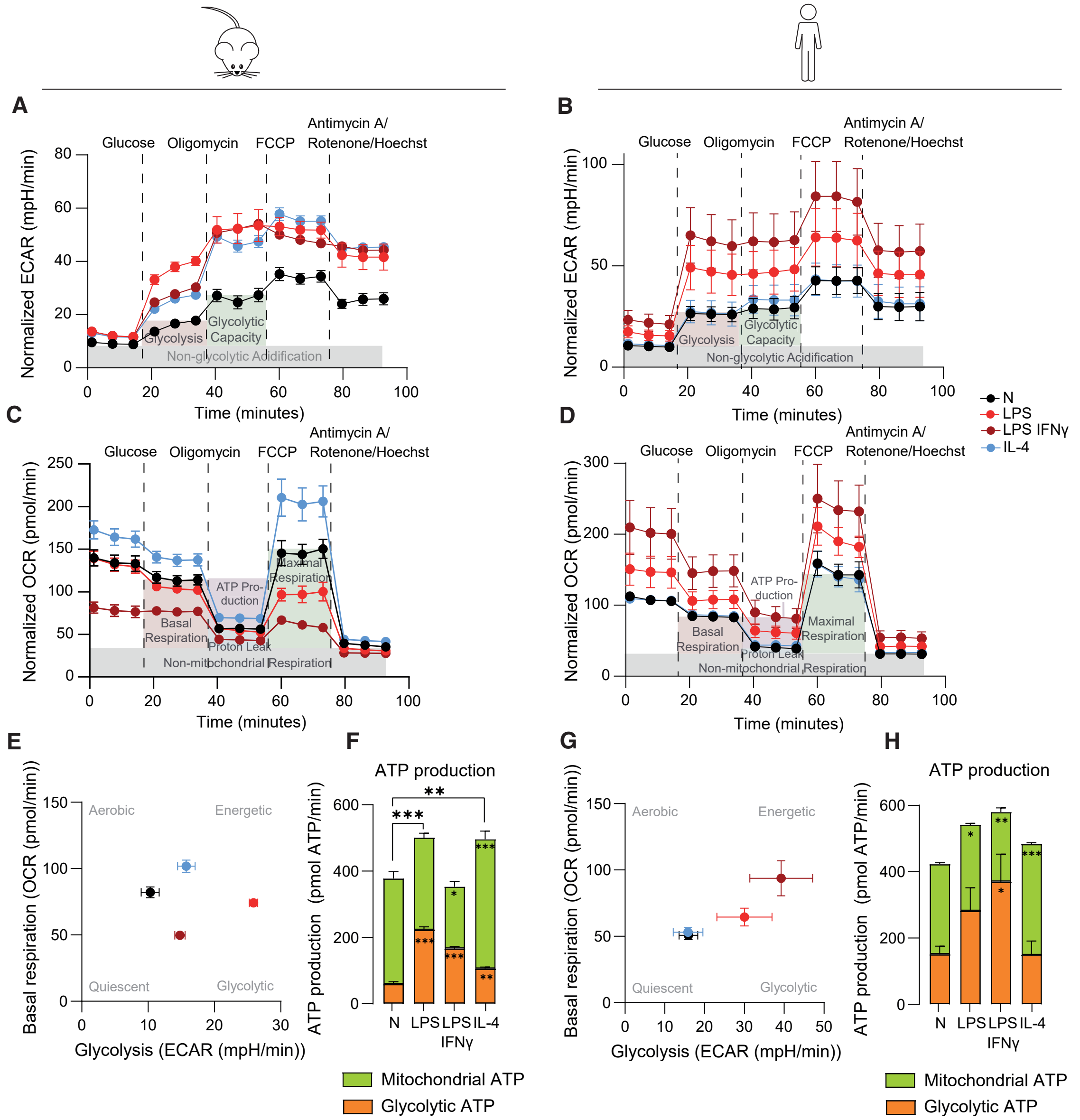

Figure 3. XF analyses of BMDMs and HMDMs yield insight into metabolic profiles of macrophages after LPS ± IFNγ and IL-4-activation

(A and B) Normalized (to relative Hoechst$^+$ objects) ECAR, with injections of glucose, oligomycin, FCCP, and antimycin A/rotenone/Hoechst for BMDMs (A) and HMDMs (B).

(C and D) Normalized (to relative Hoechst$^+$ objects) OCR with same injections as for ECAR for BMDMs (C) and HMDMs (D).

(E and G) Metabolic profiles outlining basal respiration and glycolysis for BMDMs (E) and HMDMs (G).

(F and H) Mitochondrial and glycolytic contribution to overall ATP production in BMDMs (F) and HMDMs (H); n = 6 mice or n = 6 donors were included with four to five technical replicates each. Values shown as mean ± SEM calculated from the average of technical replicates per mouse/donor. *$p < 0.05$, **$p < 0.01$, ***$p < 0.001$ by one-way ANOVA with Dunnett's post hoc test for multiple comparisons. For (F) and (H), significance on top of bar graphs indicates changes in total ATP production rate; significance within bars indicates significant differences between either the glycolytic or mitochondrial contribution to ATP production rate compared with N.

Measurement of puromycin MFI after addition of inhibitors allowed for the calculation of distinct metabolic parameters as visualized in Figure 4C:

- Glucose and mitochondrial dependency were calculated as the proportion of protein synthesis dependent on glucose oxidation and OXPHOS, respectively (Figures 4A–4C).
- Glycolytic capacity and fatty acid and/or amino acid oxidation (FAO/AAO) capacity indicate the maximum potential of cells to sustain protein synthesis when OXPHOS and glucose oxidation are inhibited, respectively (Arguello et al., 2020).

DG almost completely abolished the puromycin signal in all macrophage stimulations, indicating overall high glucose

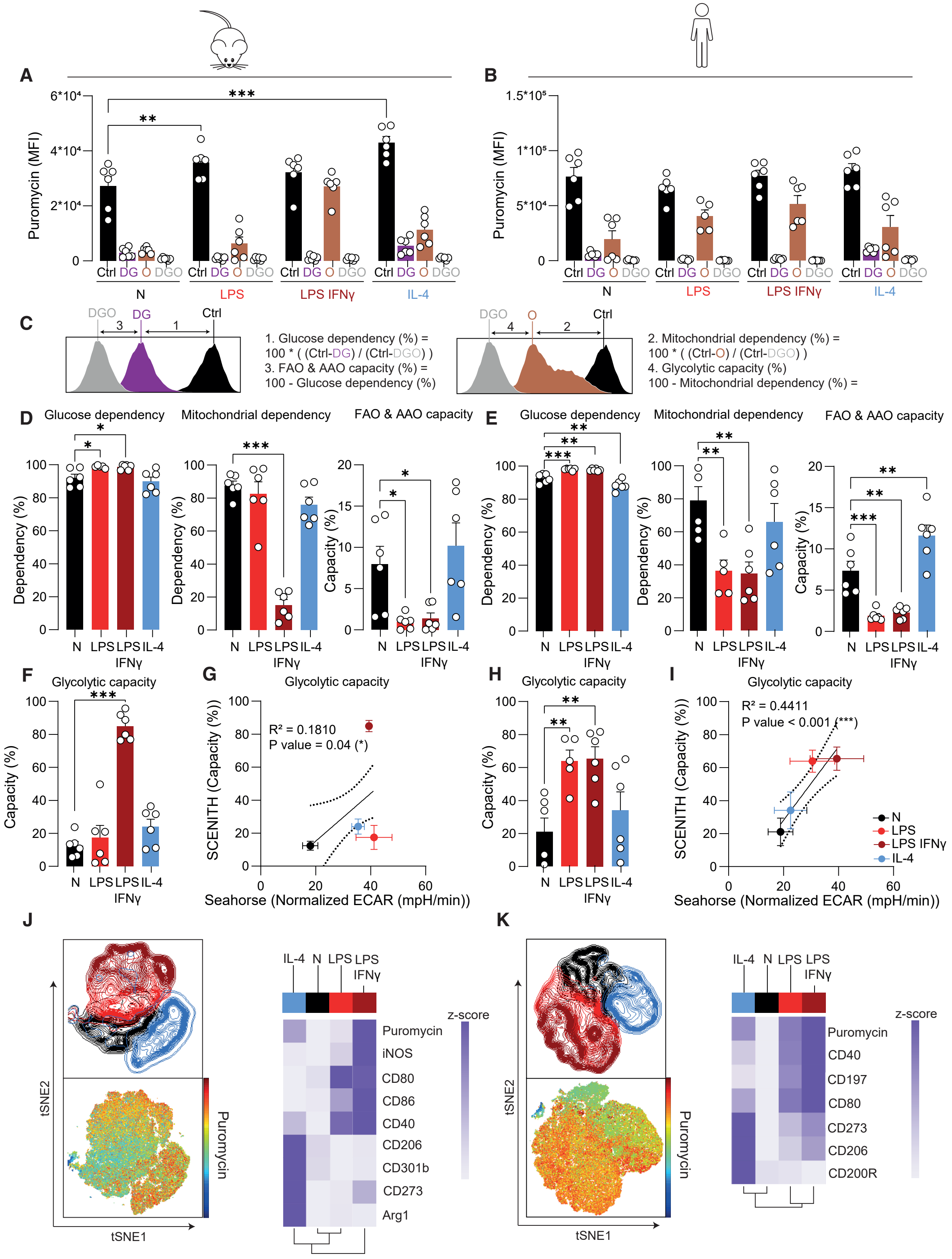

(legend on next page)

dependency (Figures 4A–4E). While M[LPS ± IFNγ] depended almost exclusively on glucose, naive and IL-4-treated BMDMs and HMDMs had the capacity to use FAO/AAO as fuel (Figures 4D and 4E). Accordingly, inhibiting mitochondrial ATP production with oligomycin (O) had the smallest effect in M[LPS + IFNγ] (Figures 4A, 4B, 4D, and 4E). Together, the SCENITH-based calculations revealed reduced mitochondrial dependency and high glycolysis in M[LPS + IFNγ] (Figures 4D, 4E, 4F, and 4H), which aligns with data obtained by XF analysis (Figures 4G and 4I).

An important advantage of SCENITH is that it can be combined with larger flow cytometry panels including, for example, M[LPS ± IFNγ]- and M[IL-4]-associated activation markers. To test the capacity of SCENITH to directly link macrophage metabolism (as measured by puromycin inhibition) to phenotype (surface markers), we performed tSNE dimensionality reduction on oligomycin-treated BMDMs and HMDMs (Figures 4J and 4K). In tSNE graphs, differentially activated macrophages cluster separately for both mouse and human, with substantial overlap between the LPS and LPS + IFNγ populations (Figures 4J and 4K). Puromycin levels of oligoymcin-treated cells were highest, and thus the least affected by oligomycin, in ($iNOS^{hi}$) $CD80^{hi}$ $CD40^{hi}$ M[LPS + IFNγ] (Figures 4J, 4K, S3E, and S3F), which in other analyses also showed to be the least dependent on mitochondria for ATP production. Although oligomycin stimulation in the SCENITH experiment can be analyzed at single-cell resolution as shown in Figures 4J and 4K, the cells need to be split into distinct wells and conditions to calculate all metabolic dependency and capacity parameters shown in the rest of Figure 4. As such, SCENITH should be regarded as cell subset analysis and not truly single-cell analysis.

Measuring incorporation of fluorescent nutrient analogs yields complementary information to XF analysis and SCENITH

To advance from SCENITH-based subset analysis to single-cell resolution, the uptake of fluorescent dyes that provide distinct insights into cellular metabolism can be measured by flow cytometry or imaging. We first titrated the dyes, followed by performing a qualitative assessment via fluorescent imaging (Figures 5A and 5B) and validation that obtained signal could be inhibited by specific inhibitors to ensure that the selected concentration was not too high (Figure S4).

2NB-DG is a fluorescent glucose analog that is used to assess glucose uptake by cells during the 30-min incubation in the presence of this dye. Flow cytometry revealed an elevated uptake of 2NB-DG in LPS-stimulated BMDMs, which correlated with the increased glycolysis as determined by XF analysis (Figure 5C). Compared with naive macrophages, LPS + IFNγ-activated BMDMs showed decreased 2NB-DG uptake, yet they consumed more glucose over a 24-h time period (Figure 2D), probably indicating that they were saturated and were no longer capable of taking up more 2NB-DG. Although HMDMs did not show differences in 2NB-DG uptake (Figure 5E), it is a single-cell approach to assess glucose uptake that significantly correlates with bulk glycolysis measurements during XF analysis (Figure 5G).

In parallel to 2NB-DG, the fluorescent BODIPY C16 can be applied as a marker to estimate fatty acid uptake by cells via flow cytometry. In mouse macrophages, fatty acid uptake was increased by IL-4 and decreased by LPS ± IFNγ (Figure 5D). This aligns with the observed FAO/AAO capacity by SCENITH and the fact that M[IL-4] macrophages show enhanced mitochondrial OXPHOS and FAO, whereas their LPS ± IFNγ-induced inflammatory counterparts do not (Figures 3C and 4D). In HMDMs, fatty acid uptake was mostly increased by macrophage activation with both LPS + IFNγ and IL-4. Whereas fatty acid uptake cannot always be directly correlated with mitochondrial oxygen consumption (Figure 5F), BODIPY C16 provides an easy cytometry-based approach to assess fatty acid uptake in single cells.

MitoTracker Green is a fluorescent mitochondrial stain that is commonly used to estimate the mitochondrial mass of cells by flow cytometry. Its signal aligns well with other mitochondrial mass measurements including mtDNA/genomic DNA ratio and mitochondrial complex immunoblotting, and in certain conditions it relates to the spare respiratory capacity of cells (Baardman et al., 2018; van der Windt et al., 2012). Here, the MitoTracker Green signal was unchanged in all BMDMs and was decreased by the different stimuli in HMDMs (Figures 5G and 5I). In this setting, no correlation was found with XF analysis-derived mitochondrial readouts. In BMDMs, the MitoTrackerGreen signal was unaffected by FCCP treatment, whereas a decrease was visible for HMDMs, indicating that MitoTracker Green staining may not be independent of mitochondrial membrane potential for all species or cell types (Figures S4A–S4D).

Tetramethylrhodamine, methyl ester (TMRM) is a red fluorescent probe that is designed to quantify changes in mitochondrial membrane potential. Such changes can relate to metabolic stress, proton leak, reverse electron transport, and ROS production (Zorova et al., 2018), and as such, TMRM signals often do not directly correlate with XF-derived mitochondrial parameters.

Figure 4. Metabolic analysis of BMDMs and HMDMs with SCENITH reveals expected macrophage activation by LPS ± IFNγ, and IL-4

(A and B) MFI of puromycin across samples treated with different inhibitors for BMDMs (A) and HMDMs (B). DG, O and DGO indicate Deoxyglucose- (DG), oligomycin- (O), or deoxyglucose + oligomycin-treated (DGO) samples.

(C) Calculations of metabolic SCENITH parameters based on puromycin MFI.

(D, E, F, and H) SCENITH parameters as calculated for mouse (D and F) and human (E and H) macrophages.

(G and I) Correlation of glycolytic capacity as measured with XF analysis with glycolytic capacity as measured with SCENITH for BMDMs (G) and HMDMs (I).

(J and K) tSNE dimensionality reduction of naive, LPS ± IFNγ-, and IL-4-treated BMDMs (J) and HMDMs (K) and clustered heatmaps showing the expression of activation markers and puromycin per stimulus.

Data are shown as mean ± SEM Each dot marks a separate mouse (n = 6) or human donor (n = 6). *$p < 0.05$, **$p < 0.01$, ***$p < 0.001$ by two-way (A and B) or ordinary one-way ANOVA(D, E, F, and H) with Dunnett's post hoc test for multiple comparisons. Correlations were fitted using a simple linear regression model (G and I).

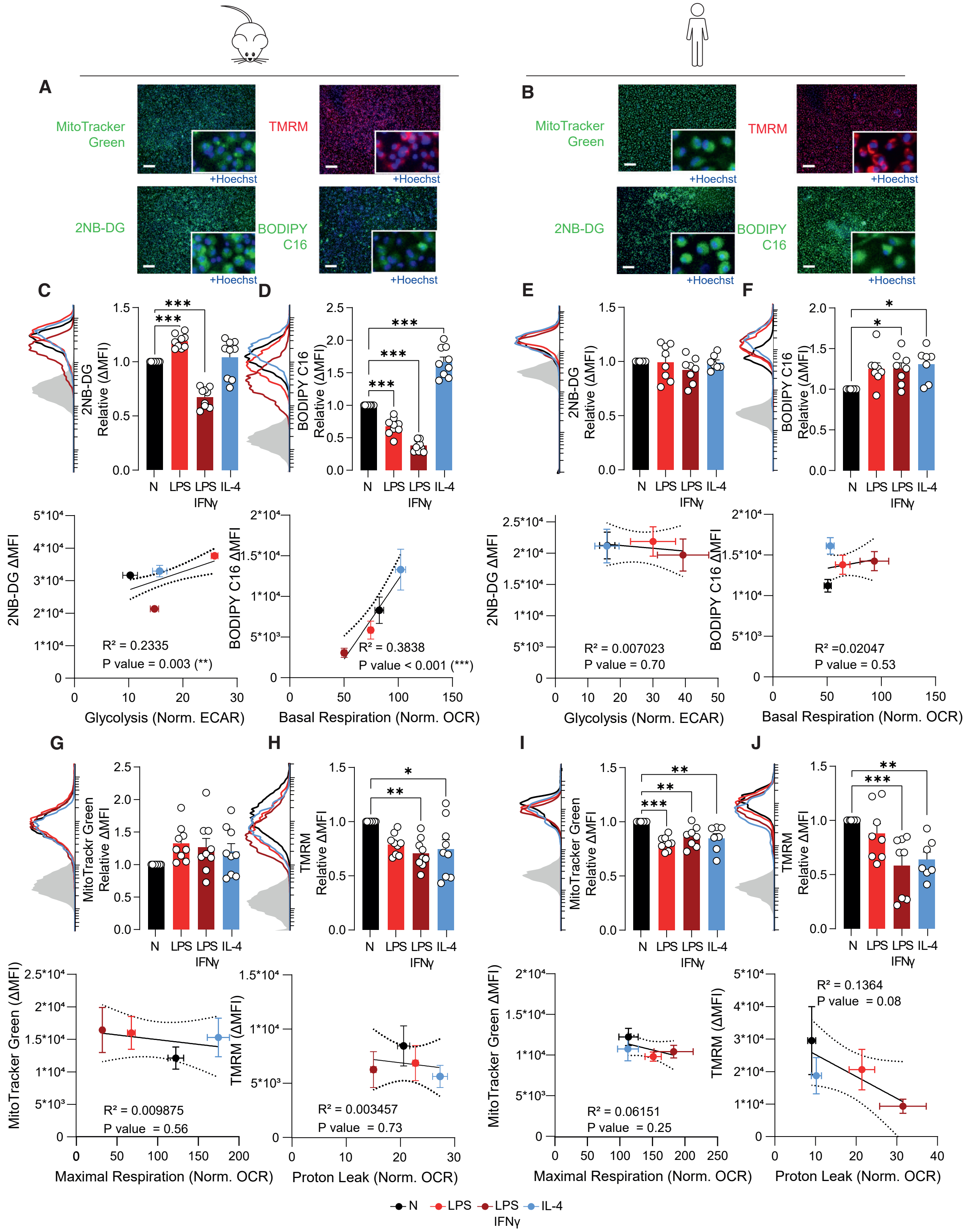

(legend on next page)

Here, we observed a drop in TMRM signal in LPS + IFNγ- and IL-4-stimulated BMDMs and HMDMs, which did not correlate with OCR-derived mitochondrial readouts (Figures 5H and 5J).

Overall, the fluorescent probes provide an accessible option to estimate metabolic changes at single-cell resolution using equipment that is present in most laboratories. For good biological interpretation and in-depth insight, these readouts should be combined with other metabolic analyses presented above or followed up by more advanced readouts described below.

Potential follow-up beyond the 96-well plate-based metabolic profiling approach

When researchers want to obtain more in-depth knowledge about the metabolic changes observed in the readouts described above, we refer to a detailed overview of the distinct possibilities (Voss et al., 2021), as well as to novel approaches providing single-cell and/or spatial resolution that are especially applicable in patient material (Artyomov and Van den Bossche, 2020). One potential follow-up option we further tested here is the use of substrate-coated plates, as this is a less established technique. However, it might provide additional insight into macrophage immunometabolism.

This technique is based on the reduction of a redox dye by NAD(P)H or $FADH_2$ production as a result of mitochondrial respiration (Figure 1). Intact cells added to carbon-substrate-coated plates (PM-M01 plates) reveal insight in their utilization of sugars. Alternatively, permeabilized cells added to plates coated with mitochondrial respiration-related carbon sources (MitoPlate S-1) reveal increased insight into mitochondrial function.

Using carbon-substrate-coated plates, we observed that intact BMDMs highly upregulated D-glucose utilization after both LPS- and IL-4-activation (Figure S5A). The maximum rate of D-glucose oxidation correlated well with glycolysis as determined by XF analysis and with 2NB-DG uptake (Figure S5B). In parallel, we used mitochondrial-substrate-coated plates to obtain information about TCA cycle and mitochondrial function in distinct macrophage subsets (Figures S5C and S5D). In agreement with the described breaks in the TCA cycle at IDH and SDH in inflammatory macrophages (Jha et al., 2015), we observed decreased oxidation of isocitrate and succinate in LPS-activated BMDMs but not HMDMs (Figures S6C and S6D). As such, the use of substrate-coated plates can be a useful follow-up method to study enzyme activity across a variety of metabolic pathways. Yet, it should be noted that this approach also yielded unexpected results that appear in contrast with published results and data obtained by more established techniques such as XF analysis. For example, upregulation of mitochondrial respiration in M[IL-4] (Figures 3C and S2C) could not be reproduced in either plate type, and alterations in FAO as observed with SCENITH (Figures 4D and 4E) could not be confirmed (Figures S6E and S6F).

Together, the techniques assessed here investigate metabolism from different angles, and all contribute to a deeper understanding of metabolic phenotypes. In the next sections, results will be discussed, followed by a consideration of strengths and limitations of each technique.

DISCUSSION

The recently increased appreciation that metabolic reprogramming is essential during innate immune responses now requires immunologists to select between different techniques and interpret data without formal training in metabolism. To help researchers entering the immunometabolism field, we have laid out an approach that integrates distinct 96-well-plate-based metabolic assays and can be combined with common immune readouts (e.g., ELISA, cytometry) (Figure 1). This allows for easy, fast, and cost-efficient pre-screening of the effect of candidate drugs (or other interventions) on immune cell metabolism, along with function, before moving toward more advanced metabolic assays. In this discussion, we describe the strengths, weaknesses, pitfalls, and caveats associated with the available assays and provide a practical workflow that guides readers through the different possibilities, ranging from basic to more advanced.

Profiling immunometabolism highlights distinct metabolic rewiring in mouse and human macrophage activation states

As an example, we applied our immunometabolic profiling platform to LPS ± IFNγ- and IL-4-stimulated BMDMs, for which metabolic reprogramming is well described (Lauterbach et al., 2019; Van den Bossche et al., 2015; Vijayan et al., 2019), and we also applied the same stimuli to HMDMs, for which metabolic processes are less well understood. Although focused on macrophages, after optimization of certain factors (detailed in Table 1) the approach can also be extended to other cells, since individual techniques have previously been applied to, for example T cells and dendritic cells (Arguello et al., 2020; Buck et al., 2016; Everts et al., 2012; Little et al., 2020; Lopes et al., 2021; Scharping et al., 2016; Thomas and Mattila, 2014).

Mouse macrophages from different (sub)strains and mouse and human macrophages cannot be directly compared due to differences in metabolism between mice (sub)strains, different

Figure 5. Uptake of fluorescent probes provides additional insight into macrophage metabolism

(A and B) Representative images of BMDM (A) and HMDM (B) staining by fluorescent dyes and uptake of fluorescent nutrient analogs as assessed by multi-mode reader. Scale bar represents 200 μm.

(C–F) Fluorescence intensity of 2NB-DG (C and E) and BODIPY C16 (D and F) uptake by BMDMs (C and D) and HMDMs (E and F) as examined by flow cytometry, correlated with relevant parameters of XF analysis.

(G–J) Fluorescence intensity of MitoTracker Green (G and I) and TMRM (H and J) analysis as examined by flow cytometry and correlations with relevant parameters of XF analysis in BMDMs (G and H) and HMDMs (I and J).

Data are shown as mean ± SEM For graphs of fluorescent probes, each dot marks a separate mouse (n = 9) or donor (n = 8). ΔMFI was calculated as MFI (median fluorescence intensity) of sample – MFI of unstained control. *$p < 0.05$, **$p < 0.01$, ***$p < 0.001$ by one-way ANOVA with Dunnett's post hoc test for multiple comparisons. Correlations were fitted using a simple linear regression model.

Table 1. Tools for 96-well-plate-based immunometabolic profiling

Methods	Assay principle	Advantages/disadvantages	Equipment
extracellular glucose/ lactate assay	estimate of glycolysis based on cumulative glucose consumption/ lactate secretion	+ simple, fast, cheap<br>– limited insight<br>– bulk analysis	absorbance (glucose) or fluorescent (lactate) plate reader
NO/arginase assay	estimates L-arginine metabolism via iNOS/arginase	+ simple, fast, cheap<br>– not all species/cell types<br>– bulk analysis	absorbance plate reader
extracellular flux analysis	measures ECAR and OCR as proxies of glycolysis and mitochondrial OXPHOS, respectively	+ parallel readouts of glycolysis and OXPHOS<br>+ adaptable injections and protocol<br>– dedicated (costly) instrument/consumables<br>– normalization needed<br>– bulk analysis, require cell purification/sorting	Seahorse XF analyzer
SCENITH	estimates metabolic capacities and dependencies by measuring changes in the level of protein synthesis	+ suitable for rare cells and complex samples<br>+ compatible with complex immune phenotyping<br>– assay principle requires active protein synthesis<br>– subset analysis rather than single cell for calculated parameters	flow cytometer
2-NBDG and BODIPY C16 uptake	fluorescent glucose analog and fluorescent fatty acid to estimate glucose or fatty acid uptake, respectively	+ simple, fast, single cell<br>– need to be validated with complementary readouts and appropriate controls to ensure correct interpretation	flow cytometer/multi-mode fluorescence imager/microscope
MitoTracker Green and TMRM (TMRE)	fluorescent mitochondrial mass and mitochondrial membrane potential indicator, respectively	+ simple, fast, single cell<br>– need to be validated with complementary readouts and appropriate controls to ensure correct interpretation	flow cytometer/multi-mode fluorescence imager/microscope

cell sources, and diverse differentiation methods. However, it is worth mentioning some exposed similarities and differences between both species before proceeding with a technical discussion and practical guidance:

(1) Both mouse and human macrophages induced glycolysis upon inflammatory activation, as measured consistently with multiple methods. Since inflammatory signaling and glycolysis are often strongly connected, the distinct readouts that estimate glycolytic function can be applied as a first and easy way to assess whether functional changes induced by pharmacological and/or genetic interventions are paralleled by metabolic rewiring.
(2) Although measuring L-arginine metabolism via iNOS or Arg1 was one of the first metabolic ways to discriminate between classical (LPS ± IFNγ) and alternative (IL-4) macrophage activation in mice (Munder et al., 1998), we here confirm it is not a valid approach to monitor *in vitro* responses in human macrophages. This is due to *NOS2* being epigenetically silenced in HMDMs and arginase usually not being regulated in human monocytes/macrophages *in vitro* (Gross et al., 2014; Thomas and Mattila, 2014).
(3) Since HMDMs do not produce NO *in vitro* (Figure 2C) (Gross et al., 2014), this could explain why they do not show the drop in mitochondrial respiration that is observed in their LPS ± IFNγ-induced NO-producing mouse counterparts (Van den Bossche et al., 2016; Vijayan et al., 2019).
(4) Notwithstanding the lack of OCR reduction in human inflammatory macrophages, they depend less on mitochondria for ATP production, and as such, this can be regarded as a commonality between both species.
(5) Fatty acids fuel mitochondrial respiration in reparative macrophages, and this supposedly anti-inflammatory metabolic feature was most apparent in IL-4-activated BMDMs in XF analysis and in HMDMs during SCENITH analysis.
(6) It should be noted that variation between human donors is higher than the variation between mice, as is also the case for other (immunological) readouts. Further increasing the number of replicates (+6) could help to reach significance in some human conditions.

Importantly, the observed metabolic rewiring was sometimes context and method dependent, as highlighted in the technical discussion. Finally, it is worth mentioning that we used macrophage colony-stimulating factor (M-CSF) to differentiate human monocytes from macrophages while they can also be differentiated with granulocyte-macrophage colony-stimulating factor (GM-CSF). The latter induces a more inflammatory state than M-CSF-differentiated macrophages (Jaguin et al., 2013; Lacey et al., 2012), and the limited research that compared both growth factors revealed that they could yield metabolic differences (Namgaladze and Brune, 2014). Dissecting the metabolic rewiring induced by different differentiation and activation factors is an avenue for future research. To address these and other

questions, our integrated immunometabolic profiling approach will be valuable to efficiently investigate many conditions simultaneously. The practical workflow presented here (summarized in Figure 1) allows starting with a fast metabolic pre-screening of a broad range of conditions before narrowing down to a selective set of conditions for comprehensive metabolic characterization with specialized high-end techniques.

Interrogation of distinct metabolic pathways by complementary techniques

In this section, we elucidate how the different methods can give complementary insights into cellular metabolism. Due to the nature and sensitivity of the readouts, some differences in results may occur, as detailed below.

Readouts of glycolysis

Extracellular glucose and lactate measurements, ECAR-derived parameters in XF analysis, SCENITH, 2NB-DG uptake, and analyzing glucose utilization with carbon-substrate-coated plates allow inferences about glycolysis.

The uptake of 2NB-DG, together with ECAR-derived glycolysis, and glucose usage as analyzed with carbon-substrate-coated plates all correlated significantly for BMDMs. For both BMDMs and HMDMs, glycolytic capacity determined by SCENITH significantly correlated with ECAR-derived glycolytic capacity. Therefore, these techniques give similar results and the choice of method can thus depend on practical considerations such as sample type or desired resolution (i.e., bulk or single cell), as discussed in the workflow below. On the other hand, glucose and lactate measurements in the supernatant can differ from ECAR-derived glycolysis or 2NB-DG uptake due to the nature of the readouts (i.e., cumulative over 24 h or a snapshot at the 24-h time point). This is illustrated in LPS + IFNγ-activated BMDMs, which showed increased glucose consumption and lactate production over the course of 24 h but did not show increased ECAR or 2NB-DG uptake after this period. Therefore, these are complementary techniques that examine cumulative changes in extracellular glucose and lactate during the stimulation versus effects on glycolysis after this period, and results may not always be identical.

Mitochondrial function

To measure mitochondrial parameters, we here showed the use of XF-derived OCR, SCENITH, MitoTracker Green, TMRM, and mitochondrial-substrate-coated plates. As expected, OCR measurements showed decreased mitochondrial respiration in inflammatory BMDMs but not HMDMs due to species differences, as discussed above. Still, mitochondrial contribution to total ATP production as calculated when taking along CO_2-based acidification in XF analysis, and mitochondrial dependence as assessed by SCENITH was decreased in both mouse and human inflammatory macrophages. Mitochondrial-substrate-coated plates confirmed this reduction in LPS-activated mouse (but not human) inflammatory macrophages. However, the substrate utilization assays appear less sensitive than XF and SCENITH, since the metabolism of the individual mitochondrial substrates was not indicative of the well-established elevated mitochondrial respiration in IL-4-activated BMDMs when analyzing intact cells with carbon-substrate-coated plates or permeabilized cells with mitochondrial-substrate-coated plates. Since XF and SCENITH yield similar results, they can be selected on the basis of other criteria.

Other parameters related to mitochondrial function include mitochondrial mass and membrane potential, measured by MitoTracker Green and TMRM, respectively. Whereas MitoTracker Green is a reliable estimate of mitochondrial mass in BMDMs, caution and additional validation are warranted for HMDMs, since staining may not be independent of mitochondrial membrane potential. Additionally, although reduced mitochondrial mass can in certain cases (such as in foam cells) explain a decrease in maximal respiration (Baardman et al., 2018), changes in respiration as seen in activated macrophages are not by definition accompanied by a change in mitochondrial mass. Similarly, changes in mitochondrial membrane potential can reflect, among others, metabolic stress and reverse electron transport (Zorova et al., 2018) and are difficult to directly relate to mitochondrial parameters. Furthermore, LPS treatment has shown both increases (Mills et al., 2016) and decreases (Yu et al., 2020) in mitochondrial membrane potential (TMRM), and is time dependent (Bauerfeld et al., 2012). Therefore, although these readouts may correlate under certain circumstances, interpretation of results can be difficult and are therefore not a first go-to assay.

Fatty acid metabolism

Readouts for fatty acid metabolism demonstrated here include SCENITH, BODIPY C16 uptake, and fatty acid utilization in mitochondrial-substrate-coated plates. SCENITH-derived FAO/AAO capacity followed the expected pattern for both BMDMs and HMDMs, although SCENITH cannot formally distinguish between FAO and AAO capacities. SCENITH-derived FAO/AAO capacity was paralleled by BODIPY C16 signal for BMDMs but not for HMDMs, indicating that uptake of fatty acids is not a direct measure of FAO. Rather, fatty acids can be stored in lipid droplets instead of being used to fuel metabolic processes (Feingold et al., 2012; Huang et al., 2014b). Mitochondrial-substrate-coated plates did not pick up differences in fatty acid metabolism between conditions. Therefore, SCENITH and BODIPY C16 uptake can give first indications about fatty acid metabolism and could be extended by a BODIPY dye staining lipid droplets (Qiu and Simon, 2016) or an adapted XF protocol allowing to probe fuel preferences (Voss et al., 2021).

While we have now described the readouts per metabolic pathway, many techniques assess parameters of several pathways in parallel. The following section addresses advantages and disadvantages for each technique, in combination with a potential practical workflow to navigate through the distinct readouts.

Practical considerations

Here, we offer practical considerations and assess strengths and limitations of each approach to provide a practical guide to using the distinct techniques (Figure 6; Table 1).

Pre-screening of metabolic alterations

Generally, the metabolic pre-screening assays are quick, cost efficient, and easy to perform and are combinable with functional assays (e.g., cytokine ELISAs and viability assays). Therefore, the pre-screening is well suited to screening many conditions

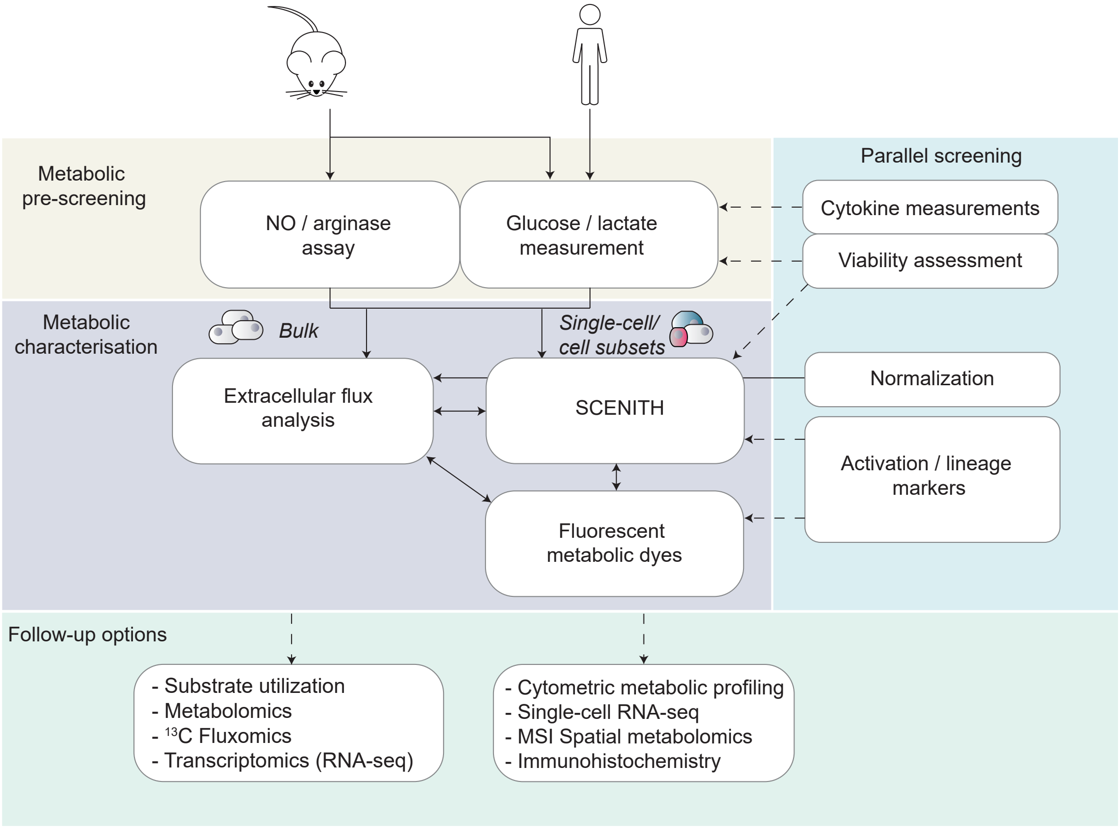

Figure 6. An actionable workflow to guide researchers from simple screening toward complex measurement of immunometabolism

Immunometabolic alterations can be pre-screened by quick and easy assays such as NO production and arginase activity in mouse macrophages and glucose consumption and lactate production in both species (yellow). Cytokine and viability measurements (green) can be performed in parallel to connect cellular metabolic changes and function. These assays can be followed up by metabolic characterization (purple) with a bulk (XF) analysis or single-cell approaches (SCENITH and fluorescent metabolic dyes). Normalization should be performed in parallel to XF analysis, and optional phenotyping can be done by adding activation and/or lineage markers to SCENITH and fluorescent metbolic dyes. Experiments can be further extended using more complex techniques (green), such as substrate utilization, metabolomics/fluxomics, RNA-seq for bulk analysis of homogeneous samples, or metabolic profiling using cytometry, single-cell RNA-seq, spatial metabolomics, or immunohistochemistry for complex samples where single-cell or spatial resolution is required. Solid lines indicate preferred workflow; dotted lines indicate optional readouts.

simultaneously. NO and arginase assays are particularly useful to profile altered L-arginine metabolism in mouse macrophages and can also be used for neutrophils, dendritic cells, and natural killer cells (Thomas and Mattila, 2014). In case NO production is altered, assessing mitochondrial OCR with XF analysis is recommended, since NO is known to affect mitochondrial function (Everts et al., 2012; Van den Bossche et al., 2016). Given the basic nature of these measurements, which does not always reflect the intricate regulation of the metabolic pathways, researchers should be aware that finding no changes in glucose, lactate, NO, or arginase levels does not automatically imply no metabolic changes. Therefore, if other indications exist to study metabolism (e.g., results of RNA-seq), more advanced metabolic readouts are a recommended next step.

XF analysis profiles glycolysis and mitochondrial function in parallel

XF analysis is currently a commonly used tool to study glycolysis and mitochondrial respiration. Besides their standard use as described in the present study, injected inhibitors can be customized to assess dependence on main fuels or activity of electron transport chain components (Salabei et al., 2014; Voss et al., 2021). Therefore, it is often a standard tool in an immunometabolism researcher's repertoire.

However, it also comes with certain disadvantages, limiting its applications. Although extremely useful for homogeneous samples of which many cells are present (such as *in vitro* stimulated cells), only the core metabolic pathways that directly result in H^+ production or oxygen consumption are represented. Additionally, analysis of complex *in vivo* samples such as tumor tissue is much more challenging. Such samples include many distinct cell populations that cannot be separately assessed without prior cell sorting. Both cell sorting and cell culturing may affect the metabolic state of cells (Binek et al., 2019; Llufrio et al., 2018; Voss et al., 2021). Generally, XF analysis requires large cell numbers in the 96-well format (typically 5,000–200,000 cells in 3–8 replicates).

Furthermore, since changes in viability, adherence, proliferation, and plating may affect XF readouts, these data need to

CellPress
OPEN ACCESS

be carefully normalized. This can be done by measuring protein content (e.g., BCA protein assay), DNA content (e.g., CyQUANT), or biomass (e.g., crystal violet) on standard (fluorescent) plate readers or by cell counts using a 96-well plate imager. We refer to a comparison for details about the specific advantages and disadvantages of distinct normalization methods for XF analysis (Kam et al., 2018).

Additionally, for further assessing viability, we recommend assays based on cell membrane integrity (such as adding a live/dead marker in flow cytometry) instead of assays based on mitochondrial activity (such as the MTT assay), since mitochondrial activity may be affected without decreasing viability.

SCENITH allows metabolic phenotyping of distinct cell subsets

When the sample to assess consists of distinct cell subsets and when isolation of rare populations yields cell numbers too low for XF analysis, SCENITH can be used to evaluate metabolism by using just one fluorescent channel in flow cytometry. It can provide additional phenotypic (and metabolic) insights when combined with antibodies against immune activation markers (and key metabolic mediators) in larger cytometry panels. Similarly to XF analysis, the inhibitors used can also be extended or customized to assess different metabolic dependencies.

Although a range of cell types was tested in the original publication, situations may exist for which protein synthesis is not directly related to ATP synthesis, e.g., in quiescent stem cells (Arguello et al., 2020). Furthermore, since the metabolic parameters are calculated from samples treated with inhibitors in parallel rather than from a single sample, metabolic parameters cannot be calculated for each single cell but rather for cell subsets. Additionally, combining SCENITH with large panels can become costly, since each sample needs to be split, stained, and measured in parallel in order to accommodate the different inhibitors.

Fluorescent metabolic probes enable assessing metabolic features at single-cell resolution

Fluorescent metabolic probes can be measured by flow cytometry or microscopy to provide single-cell resolution and are practical when no XF analyzer is available. Flow cytometry allows for easier quantification of data but lacks the option for visual information. Although metabolic dyes can in principle be combined into larger flow cytometry panels, this is practically limited due to protocol incompatibilities (e.g., staining temperature and duration, and not all dyes are fixable). Furthermore, care needs to be taken when metabolic properties are assessed solely by fluorescent labeling, as it was previously shown that 2NB-DG staining yielded substantially different results compared with more established glucose transport assays (D'Souza et al., 2021; Sinclair et al., 2020). This illustrates the importance of proper controls to ensure that dyes are taken up specifically. Dyes should also be titrated to prevent unspecific staining of cellular compartments. Additionally, MitoTracker Green signal should be checked for independence of mitochondrial membrane potential to reliably estimate mitochondrial mass, since this may be species or cell type dependent. Finally, interpretation of results may be difficult due lack of robustness and timing- and context-dependent effects on mitochondrial function, particularly for MitoTracker Green and TMRM.

Next to the fluorescent probes measured here, a range of other fluorescent dyes and analyses exist to analyze metabolic alterations in response to specific treatments. Examples of other probes are mitoSOX for the measurement of mitochondrial ROS or propidium iodide for the analysis of cell cycle distribution. Additionally, a confocal microscope or multi-mode reader with high resolution can be used to reveal visual differences in subcellular localization or mitochondrial fragmentation (Little et al., 2020).

The toolbox as discussed here gives an overview of metabolic alterations occurring after compound treatment and can help to narrow down stimuli of interest. These can be investigated in more detail with more complex techniques, which will be discussed briefly in the following section. For more details we refer the reader to recent reviews (Artyomov and Van den Bossche, 2020; Voss et al., 2021).

Conclusion and practical guidance

We here present an integrated 96-well-plate-based approach to screening metabolic alterations. We recommend starting with quick and easy pre-screening methods such as NO, arginase, glucose, and lactate assays before continuing with XF analysis for bulk samples, SCENITH for subset analysis of complex samples, or fluorescent dyes for single-cell resolution (Figure 6). This approach serves to efficiently analyze metabolic alterations and narrow down conditions of interest before complex and costly follow up techniques such as (single-cell) transcriptomics or (spatial) metabolomics are applied.

Limitations of the study

It should be noted that the readouts within the toolbox provide a good estimation of the immunometabolic state of macrophages but that more dedicated high-end follow-up studies can be required to obtain more in-depth insight. One potential follow-up technique that we here illustrate is substrate utilization analysis for intact and permeabilized cells, which facilitates the screening of fuel preference in homogeneous *in vitro* cultured cells. Due to the range of different substrates, a greater variety of metabolic pathways can be assessed in extensive detail.

Carbon-substrate-coated plates can measure substrate utilization in intact cells, but the assessment of mitochondria-specific substrate utilization requires cell permeabilization. This results in a rather artificial system due to the lack of feedback and inhibition by other metabolites, the inability to regulate cellular substrate uptake, and because of *ad libitum* access to substrates by mitochondria. For these reasons, the assessed substrate oxidation reflects theoretical enzyme activity but does not reflect the physiological metabolic pathway. Although this technique has been applied in other immune cells (Kalvala et al., 2019; Thwe et al., 2019; Zhang et al., 2021), it has not previously been used in cultured macrophages *in vitro*. In combination with the limited overlap with the other techniques tested here, it makes this technique a potential follow-up tool when additional validation is included.

Transcriptomic and metabolomic measurements can provide additional insight into metabolic changes. The acquired data

may be complementary and can be integrated (Jha et al., 2015) but may seem contradictory when enzyme regulation takes place on different levels. For example, post-transcriptional or post-translational modifications are not included in transcriptomic data, and differences in gene expression do not always correlate with active translation into protein and enzyme activity. We refer the reader to recent reviews (Artyomov and Van den Bossche, 2020; Voss et al., 2021) for a further discussion of (single-cell) transcriptomics, metabolomics, and their integration and other single-cell techniques to study cellular metabolism such as cytometry-based metabolic panels (Ahl et al., 2020; Hartmann et al., 2021; Levine et al., 2021). These methods will greatly enhance insight into metabolic alterations upon treatment with compounds of interest, but they do require more specialized machines and can complicate data analysis.

In the metabolic analysis of complex *in vivo* samples, spatial and temporal knowledge of metabolic processes are of major additional value (Artyomov and Van den Bossche, 2020; Murphy and O'Neill, 2020). Therefore, imaging-based techniques (Miller et al., 2017; Palmer et al., 2017) such as GeoMX (Farren et al., 2020) or MIBI-TOF (Hartmann and Bendall, 2020; Keren et al., 2019) have been developed that combine transcriptomic, metabolic, and functional readouts with spatial information. Also, approaches that assess subcellular metabolism are important, since metabolites accumulate to a different extent in diverse subcellular locations and may affect (signaling) targets differently. This was, for example, indicated for acyl intermediates and their ability to modify proteins (Bambouskova et al., 2021; Murphy and O'Neill, 2020). Additionally, to prove whether a metabolic change is a cause or a consequence of phenotypic alterations, it is desirable to assess temporal aspects. This can be implemented, e.g., by repeated ^{13}C-metabolomic flux measurements in combination with phenotypic assessment or the analysis of pseudo-time in single-cell RNA-seq. By including spatiotemporal approaches in immunometabolic research, discrepancies in the literature may be resolved. Finally, causality needs to be demonstrated by targeting metabolic enzymes with genetic or pharmacological tools, as described previously in more detail (Voss et al., 2021).

STAR★METHODS

Detailed methods are provided in the online version of this paper and include the following:

- KEY RESOURCES TABLE
- RESOURCE AVAILABILITY
  - ○ Lead contact
  - ○ Materials availability
  - ○ Data and code availability
- EXPERIMENTAL MODEL AND SUBJECT DETAILS
  - ○ Bone marrow isolation and BMDM culture
  - ○ Monocyte isolation and HMDM culture
- METHOD DETAILS
  - ○ NO production and arginase activity assay
  - ○ Glucose consumption assay
  - ○ Lactate production assay
  - ○ Extracellular flux analysis
  - ○ SCENITH and flow cytometry and analysis
  - ○ Fluorescent metabolic dyes
  - ○ Mitochondrial functional substrate assay
- QUANTIFICATION AND STATISTICAL ANALYSIS

SUPPLEMENTAL INFORMATION

Supplemental information can be found online at https://doi.org/10.1016/j.crmeth.2022.100192

ACKNOWLEDGMENTS

We thank Sam de Decker, Enrico Tatti, and Barry Bochner for supplying all materials to perform the Biolog mitochondrial-substrate-coated and carbon-substrate-coated assays, their help with setting up the assays and interpreting the results, and their helpful comments on the manuscript. We further thank Amber Eggert for optimizing the Hoechst cell-counting protocol on the Cytation five and Karl Harber for proofreading the manuscript. We thank ANR and Inserm Transfert for the ANR-20-CE-CE14-0028-01 and CoPoC MAT-PI-17493-A-04 grants to R.J.A. J.V.d.B. received a Netherlands Heart Foundation senior fellowship (2017T048). Additionally, J.V.d.B. received an Enw-Klein-1 grant from NWO (OCENW.KLEIN.268) and a consortia grant from European Research Area network on Cardiovascular Diseases (ERA-CVD 2019T108). Last, we acknowledge support from Cancer Center Amsterdam.

AUTHOR CONTRIBUTIONS

S.V. and K.d.G. contributed equally to this work; order of authorship was decided by flipping a coin. Conceptualization, J.V.d.B., S.V., and K.d.G; methodology, S.V., K.d.G., F.G., and R.A.; formal analysis, S.V. and K.d.G.; investigation, S.V., K.d.G., F.G., and X.v.D.; visualization, S.V. and K.d.G.; writing – review & editing, S.V., K.d.G., F.G., X.v.D., R.A., and J.V.d.B.; funding acquisition, J.V.d.B. All authors read and approved the final manuscript.

DECLARATION OF INTERESTS

The authors declare no competing interests. There are restrictions to the commercial use of SCENITH due to a pending patent application (PCT/EP2020/060,486).

Received: June 3, 2021
Revised: December 23, 2021
Accepted: March 7, 2022
Published: March 28, 2022

REFERENCES

Ahl, P.J., Hopkins, R.A., Xiang, W.W., Au, B., Kaliaperumal, N., Fairhurst, A.M., and Connolly, J.E. (2020). Met-Flow, a strategy for single-cell metabolic analysis highlights dynamic changes in immune subpopulations. Commun. Biol. *3*, 305. https://doi.org/10.1038/s42003-020-1027-9.

Arguello, R.J., Combes, A.J., Char, R., Gigan, J.P., Baaziz, A.I., Bousiquot, E., Camosseto, V., Samad, B., Tsui, J., Yan, P., et al. (2020). SCENITH: a flow cytometry-based method to functionally profile energy metabolism with single-cell resolution. Cell Metab. *32*, 1063–1075.e67. https://doi.org/10.1016/j.cmet.2020.11.007.

Artyomov, M.N., and Van den Bossche, J. (2020). Immunometabolism in the single-cell era. Cell Metab. *32*, 710–725. https://doi.org/10.1016/j.cmet.2020.09.013.

Baardman, J., Verberk, S.G.S., Prange, K.H.M., van Weeghel, M., van der Velden, S., Ryan, D.G., Wust, R.C.I., Neele, A.E., Speijer, D., Denis, S.W., et al. (2018). A defective pentose phosphate pathway reduces inflammatory macrophage responses during hypercholesterolemia. Cell Rep. *25*, 2044–2052.e45. https://doi.org/10.1016/j.celrep.2018.10.092.

CellPress
OPEN ACCESS

Bailey, J.D., Diotallevi, M., Nicol, T., McNeill, E., Shaw, A., Chuaiphichai, S., Hale, A., Starr, A., Nandi, M., Stylianou, E., et al. (2019). Nitric oxide modulates metabolic remodeling in inflammatory macrophages through TCA cycle regulation and itaconate accumulation. Cell Rep. *28*, 218–230.e17. https://doi.org/10.1016/j.celrep.2019.06.018.

Bambouskova, M., Potuckova, L., Paulenda, T., Kerndl, M., Mogilenko, D.A., Lizotte, K., Swain, A., Hayes, S., Sheldon, R.D., Kim, H., et al. (2021). Itaconate confers tolerance to late NLRP3 inflammasome activation. Cell Rep. *34*, 108756. https://doi.org/10.1016/j.celrep.2021.108756.

Bauerfeld, C.P., Rastogi, R., Pirockinaite, G., Lee, I., Huttemann, M., Monks, B., Birnbaum, M.J., Franchi, L., Nunez, G., and Samavati, L. (2012). TLR4-mediated AKT activation is MyD88/TRIF dependent and critical for induction of oxidative phosphorylation and mitochondrial transcription factor A in murine macrophages. J. Immunol. *188*, 2847–2857. https://doi.org/10.4049/jimmunol.1102157.

Binek, A., Rojo, D., Godzien, J., Ruperez, F.J., Nunez, V., Jorge, I., Ricote, M., Vazquez, J., and Barbas, C. (2019). Flow cytometry has a significant impact on the cellular metabolome. J. Proteome Res. *18*, 169–181. https://doi.org/10.1021/acs.jproteome.8b00472.

Buck, M.D., O'Sullivan, D., Klein Geltink, R.I., Curtis, J.D., Chang, C.H., Sanin, D.E., Qiu, J., Kretz, O., Braas, D., van der Windt, G.J., et al. (2016). Mitochondrial dynamics controls T cell fate through metabolic programming. Cell *166*, 63–76. https://doi.org/10.1016/j.cell.2016.05.035.

Chan, D.M., Rao, R., Huang, F., and Canny, J.F. (2019). GPU accelerated t-distributed stochastic neighbor embedding. J. Parallel Distribut. Comput. *131*, 1–13. https://doi.org/10.1016/j.jpdc.2019.04.008.

D'Souza, L.J., Wright, S.H., and Bhattacharya, D. (2021). Genetic evidence that uptake of the fluorescent analog 2NBDG occurs independently of known glucose transporters. Preprint at bioRxiv. https://doi.org/10.1101/2021.12.13.472409.

Everts, B., Amiel, E., van der Windt, G.J., Freitas, T.C., Chott, R., Yarasheski, K.E., Pearce, E.L., and Pearce, E.J. (2012). Commitment to glycolysis sustains survival of NO-producing inflammatory dendritic cells. Blood *120*, 1422–1431. https://doi.org/10.1182/blood-2012-03-419747.

Farren, M.R., Sayegh, L., Ware, M.B., Chen, H.R., Gong, J., Liang, Y., Krasinskas, A., Maithel, S.K., Zaidi, M., Sarmiento, J.M., et al. (2020). Immunologic alterations in the pancreatic cancer microenvironment of patients treated with neoadjuvant chemotherapy and radiotherapy. JCI Insight *5*. https://doi.org/10.1172/jci.insight.130362.

Feingold, K.R., Shigenaga, J.K., Kazemi, M.R., McDonald, C.M., Patzek, S.M., Cross, A.S., Moser, A., and Grunfeld, C. (2012). Mechanisms of triglyceride accumulation in activated macrophages. J. Leukoc. Biol. *92*, 829–839. https://doi.org/10.1189/jlb.1111537.

Freemerman, A.J., Johnson, A.R., Sacks, G.N., Milner, J.J., Kirk, E.L., Troester, M.A., Macintyre, A.N., Goraksha-Hicks, P., Rathmell, J.C., and Makowski, L. (2014). Metabolic reprogramming of macrophages: glucose transporter 1 (GLUT1)-mediated glucose metabolism drives a proinflammatory phenotype. J. Biol. Chem. *289*, 7884–7896. https://doi.org/10.1074/jbc.M113.522037.

Galli, G., and Saleh, M. (2020). Immunometabolism of macrophages in bacterial infections. Front. Cell Infect. Microbiol. *10*, 607650. https://doi.org/10.3389/fcimb.2020.607650.

Gross, T.J., Kremens, K., Powers, L.S., Brink, B., Knutson, T., Domann, F.E., Philibert, R.A., Milhem, M.M., and Monick, M.M. (2014). Epigenetic silencing of the human NOS2 gene: rethinking the role of nitric oxide in human macrophage inflammatory responses. J. Immunol. *192*, 2326–2338. https://doi.org/10.4049/jimmunol.1301758.

Harber, K.J., de Goede, K.E., Verberk, S.G.S., Meinster, E., de Vries, H.E., van Weeghel, M., de Winther, M.P.J., and Van den Bossche, J. (2020). Succinate is an inflammation-induced immunoregulatory metabolite in macrophages. Metabolites *10*. https://doi.org/10.3390/metabo10090372.

Hartmann, F.J., and Bendall, S.C. (2020). Immune monitoring using mass cytometry and related high-dimensional imaging approaches. Nat. Rev. Rheumatol. *16*, 87–99. https://doi.org/10.1038/s41584-019-0338-z.

Hartmann, F.J., Mrdjen, D., McCaffrey, E., Glass, D.R., Greenwald, N.F., Bharadwaj, A., Khair, Z., Verberk, S.G.S., Baranski, A., Baskar, R., et al. (2021). Single-cell metabolic profiling of human cytotoxic T cells. Nat. Biotechnol. *39*, 186–197. https://doi.org/10.1038/s41587-020-0651-8.

Huang, S.C., Everts, B., Ivanova, Y., O'Sullivan, D., Nascimento, M., Smith, A.M., Beatty, W., Love-Gregory, L., Lam, W.Y., O'Neill, C.M., et al. (2014a). Cell-intrinsic lysosomal lipolysis is essential for alternative activation of macrophages. Nat. Immunol. *15*, 846–855. https://doi.org/10.1038/ni.2956.

Huang, Y.L., Morales-Rosado, J., Ray, J., Myers, T.G., Kho, T., Lu, M., and Munford, R.S. (2014b). Toll-like receptor agonists promote prolonged triglyceride storage in macrophages. J. Biol. Chem. *289*, 3001–3012. https://doi.org/10.1074/jbc.M113.524587.

Jaguin, M., Houlbert, N., Fardel, O., and Lecureur, V. (2013). Polarization profiles of human M-CSF-generated macrophages and comparison of M1-markers in classically activated macrophages from GM-CSF and M-CSF origin. Cell Immunol. *281*, 51–61. https://doi.org/10.1016/j.cellimm.2013.01.010.

Jha, A.K., Huang, S.C., Sergushichev, A., Lampropoulou, V., Ivanova, Y., Loginicheva, E., Chmielewski, K., Stewart, K.M., Ashall, J., Everts, B., et al. (2015). Network integration of parallel metabolic and transcriptional data reveals metabolic modules that regulate macrophage polarization. Immunity *42*, 419–430. https://doi.org/10.1016/j.immuni.2015.02.005.

Kalvala, A., Wallet, P., Yang, L., Wang, C., Li, H., Nam, A., Nathan, A., Mambetsariev, I., Poroyko, V., Gao, H., et al. (2019). Phenotypic switching of naive T cells to immune-suppressive treg-like cells by mutant KRAS. J. Clin. Med. *8*. https://doi.org/10.3390/jcm8101726.

Kam, Y., Rogers, G.W., Jastromb, N., and Dranka, B.P. (2018). Methods and strategies for normalizing XF metabolic data to cellular parameters. https://www.agilent.com/cs/library/technicaloverviews/public/Methods_and_Strategies_for_Normalizing_Tech_Overview_022118.pdf.

Keren, L., Bosse, M., Thompson, S., Risom, T., Vijayaragavan, K., McCaffrey, E., Marquez, D., Angoshtari, R., Greenwald, N.F., Fienberg, H., et al. (2019). MIBI-TOF: a multiplexed imaging platform relates cellular phenotypes and tissue structure. Sci. Adv. *5*, eaax5851. https://doi.org/10.1126/sciadv.aax5851.

Koenis, D.S., Medzikovic, L., van Loenen, P.B., van Weeghel, M., Huveneers, S., Vos, M., Evers-van Gogh, I.J., Van den Bossche, J., Speijer, D., Kim, Y., et al. (2018). Nuclear receptor Nur77 limits the macrophage inflammatory response through transcriptional reprogramming of mitochondrial metabolism. Cell Rep. *24*, 2127–2140.e27. https://doi.org/10.1016/j.celrep.2018.07.065.

Lacey, D.C., Achuthan, A., Fleetwood, A.J., Dinh, H., Roiniotis, J., Scholz, G.M., Chang, M.W., Beckman, S.K., Cook, A.D., and Hamilton, J.A. (2012). Defining GM-CSF- and macrophage-CSF-dependent macrophage responses by in vitro models. J. Immunol. *188*, 5752–5765. https://doi.org/10.4049/jimmunol.1103426.

Lachmandas, E., Boutens, L., Ratter, J.M., Hijmans, A., Hooiveld, G.J., Joosten, L.A., Rodenburg, R.J., Fransen, J.A., Houtkooper, R.H., van Crevel, R., et al. (2016). Microbial stimulation of different Toll-like receptor signalling pathways induces diverse metabolic programmes in human monocytes. Nat. Microbiol. *2*, 16246. https://doi.org/10.1038/nmicrobiol.2016.246.

Lampropoulou, V., Sergushichev, A., Bambouskova, M., Nair, S., Vincent, E.E., Loginicheva, E., Cervantes-Barragan, L., Ma, X., Huang, S.C., Griss, T., et al. (2016). Itaconate links inhibition of succinate dehydrogenase with macrophage metabolic remodeling and regulation of inflammation. Cell Metab. *24*, 158–166. https://doi.org/10.1016/j.cmet.2016.06.004.

Lauterbach, M.A., Hanke, J.E., Serefidou, M., Mangan, M.S.J., Kolbe, C.C., Hess, T., Rothe, M., Kaiser, R., Hoss, F., Gehlen, J., et al. (2019). Toll-like receptor signaling rewires macrophage metabolism and promotes histone acetylation via ATP-citrate lyase. Immunity *51*, 997–1011.e17. https://doi.org/10.1016/j.immuni.2019.11.009.

Levine, L.S., Hiam-Galvez, K.J., Marquez, D.M., Tenvooren, I., Madden, M.Z., Contreras, D.C., Dahunsi, D.O., Irish, J.M., Oluwole, O.O., Rathmell, J.C., and Spitzer, M.H. (2021). Single-cell analysis by mass cytometry reveals metabolic states of early-activated CD8(+) T cells during the primary immune response. Immunity *54*, 829–844.e25. https://doi.org/10.1016/j.immuni.2021.02.018.

Lim, A.R., Rathmell, W.K., and Rathmell, J.C. (2020). The tumor microenvironment as a metabolic barrier to effector T cells and immunotherapy. Elife *9*, e55185.

Little, A.C., Kovalenko, I., Goo, L.E., Hong, H.S., Kerk, S.A., Yates, J.A., Purohit, V., Lombard, D.B., Merajver, S.D., and Lyssiotis, C.A. (2020). High-content fluorescence imaging with the metabolic flux assay reveals insights into mitochondrial properties and functions. Commun. Biol. *3*, 271. https://doi.org/10.1038/s42003-020-0988-z.

Llufrio, E.M., Wang, L., Naser, F.J., and Patti, G.J. (2018). Sorting cells alters their redox state and cellular metabolome. Redox Biol. *16*, 381–387. https://doi.org/10.1016/j.redox.2018.03.004.

Lopes, N., McIntyre, C., Martin, S., Raverdeau, M., Sumaria, N., Kohlgruber, A.C., Fiala, G.J., Agudelo, L.Z., Dyck, L., Kane, H., et al. (2021). Distinct metabolic programs established in the thymus control effector functions of gammadelta T cell subsets in tumor microenvironments. Nat. Immunol. *22*, 179–192. https://doi.org/10.1038/s41590-020-00848-3.

Makowski, L., Chaib, M., and Rathmell, J.C. (2020). Immunometabolism: from basic mechanisms to translation. Immunological Rev. *295*, 5–14.

Martinez, F.O., Helming, L., Milde, R., Varin, A., Melgert, B.N., Draijer, C., Thomas, B., Fabbri, M., Crawshaw, A., Ho, L.P., et al. (2013). Genetic programs expressed in resting and IL-4 alternatively activated mouse and human macrophages: similarities and differences. Blood *121*, e57–e69. https://doi.org/10.1182/blood-2012-06-436212.

Miller, A., Nagy, C., Knapp, B., Laengle, J., Ponweiser, E., Groeger, M., Starkl, P., Bergmann, M., Wagner, O., and Haschemi, A. (2017). Exploring metabolic configurations of single cells within complex tissue microenvironments. Cell Metab. *26*, 788–800.e86. https://doi.org/10.1016/j.cmet.2017.08.014.

Mills, E.L., Kelly, B., Logan, A., Costa, A.S.H., Varma, M., Bryant, C.E., Tourlomousis, P., Dabritz, J.H.M., Gottlieb, E., Latorre, I., et al. (2016). Succinate dehydrogenase supports metabolic repurposing of mitochondria to drive inflammatory macrophages. Cell *167*, 457–470.e13. https://doi.org/10.1016/j.cell.2016.08.064.

Mills, E.L., and O'Neill, L.A. (2016). Reprogramming mitochondrial metabolism in macrophages as an anti-inflammatory signal. Eur. J. Immunol. *46*, 13–21. https://doi.org/10.1002/eji.201445427.

Munder, M., Eichmann, K., and Modolell, M. (1998). Alternative metabolic states in murine macrophages reflected by the nitric oxide synthase/arginase balance: competitive regulation by CD4+ T cells correlates with Th1/Th2 phenotype. J. Immunol. *160*, 5347–5354.

Murphy, M.P., and O'Neill, L.A.J. (2020). How should we talk about metabolism? Nat. Immunol. *21*, 713–715. https://doi.org/10.1038/s41590-020-0691-8.

Murray, P.J., Allen, J.E., Biswas, S.K., Fisher, E.A., Gilroy, D.W., Goerdt, S., Gordon, S., Hamilton, J.A., Ivashkiv, L.B., Lawrence, T., et al. (2014). Macrophage activation and polarization: nomenclature and experimental guidelines. Immunity *41*, 14–20. https://doi.org/10.1016/j.immuni.2014.06.008.

Namgaladze, D., and Brune, B. (2014). Fatty acid oxidation is dispensable for human macrophage IL-4-induced polarization. Biochim. Biophys. Acta. *1841*, 1329–1335. https://doi.org/10.1016/j.bbalip.2014.06.007.

Palmer, A., Phapale, P., Chernyavsky, I., Lavigne, R., Fay, D., Tarasov, A., Kovalev, V., Fuchser, J., Nikolenko, S., Pineau, C., et al. (2017). FDR-controlled metabolite annotation for high-resolution imaging mass spectrometry. Nat. Methods *14*, 57–60. https://doi.org/10.1038/nmeth.4072.

Qiu, B., and Simon, M.C. (2016). BODIPY 493/503 staining of neutral lipid droplets for microscopy and quantification by flow cytometry. Bio Protoc. *6*. https://doi.org/10.21769/BioProtoc.1912.

Rattigan, K.M., Pountain, A.W., Regnault, C., Achcar, F., Vincent, I.M., Goodyear, C.S., and Barrett, M.P. (2018). Metabolomic profiling of macrophages determines the discrete metabolomic signature and metabolomic interactome triggered by polarising immune stimuli. PLoS One *13*, e0194126. https://doi.org/10.1371/journal.pone.0194126.

Romero, N., Swain, P., Neilson, A., and Dranka, B.P. (2017). Improving Quantification of cellular glycolytic rate using agilent seahorse XF technology. https://seahorseinfo.agilent.com/acton/fs/blocks/showLandingPage/a/10967/p/p-00ca/t/page/fm/1.

Salabei, J.K., Gibb, A.A., and Hill, B.G. (2014). Comprehensive measurement of respiratory activity in permeabilized cells using extracellular flux analysis. Nat. Protoc. *9*, 421–438. https://doi.org/10.1038/nprot.2014.018.

Scharping, N.E., Menk, A.V., Moreci, R.S., Whetstone, R.D., Dadey, R.E., Watkins, S.C., Ferris, R.L., and Delgoffe, G.M. (2016). The tumor microenvironment represses T cell mitochondrial biogenesis to drive intratumoral T cell metabolic insufficiency and dysfunction. Immunity *45*, 701–703. https://doi.org/10.1016/j.immuni.2016.08.009.

Sinclair, L.V., Barthelemy, C., and Cantrell, D.A. (2020). Single cell glucose uptake assays: a cautionary tale. Immunometabolism *2*, e200029. https://doi.org/10.20900/immunometab20200029.

Tavakoli, S., Zamora, D., Ullevig, S., and Asmis, R. (2013). Bioenergetic profiles diverge during macrophage polarization: implications for the interpretation of 18F-FDG PET imaging of atherosclerosis. J. Nucl. Med. *54*, 1661–1667. https://doi.org/10.2967/jnumed.112.119099.

Thomas, A.C., and Mattila, J.T. (2014). "Of mice and men": arginine metabolism in macrophages. Front. Immunol. *5*, 479. https://doi.org/10.3389/fimmu.2014.00479.

Thwe, P.M., Pelgrom, L.R., Cooper, R., Beauchamp, S., Reisz, J.A., D'Alessandro, A., Everts, B., and Amiel, E. (2019). Cell-Intrinsic glycogen metabolism supports early glycolytic reprogramming required for dendritic cell immune responses. Cell Metab. *30*, 225. https://doi.org/10.1016/j.cmet.2019.05.017.

Van den Bossche, J., Baardman, J., and de Winther, M.P. (2015). Metabolic characterization of polarized M1 and M2 bone marrow-derived macrophages using real-time extracellular flux analysis. J. Visual. Exp. e53424 https://doi.org/10.3791/53424.

Van den Bossche, J., Baardman, J., Otto, N.A., van der Velden, S., Neele, A.E., van den Berg, S.M., Luque-Martin, R., Chen, H.J., Boshuizen, M.C., Ahmed, M., et al. (2016). Mitochondrial dysfunction prevents repolarization of inflammatory macrophages. Cell Rep. *17*, 684–696. https://doi.org/10.1016/j.celrep.2016.09.008.

Van den Bossche, J., O'Neill, L.A., and Menon, D. (2017). Macrophage immunometabolism: where are we (going)? Trends Immunol. *38*, 395–406. https://doi.org/10.1016/j.it.2017.03.001.

van der Windt, G.J., Everts, B., Chang, C.H., Curtis, J.D., Freitas, T.C., Amiel, E., Pearce, E.J., and Pearce, E.L. (2012). Mitochondrial respiratory capacity is a critical regulator of CD8+ T cell memory development. Immunity *36*, 68–78. https://doi.org/10.1016/j.immuni.2011.12.007.

Vats, D., Mukundan, L., Odegaard, J.I., Zhang, L., Smith, K.L., Morel, C.R., Wagner, R.A., Greaves, D.R., Murray, P.J., and Chawla, A. (2006). Oxidative metabolism and PGC-1beta attenuate macrophage-mediated inflammation. Cell Metab. *4*, 13–24. https://doi.org/10.1016/j.cmet.2006.05.011.

Vijayan, V., Pradhan, P., Braud, L., Fuchs, H.R., Gueler, F., Motterlini, R., Foresti, R., and Immenschuh, S. (2019). Human and murine macrophages exhibit differential metabolic responses to lipopolysaccharide - a divergent role for glycolysis. Redox Biol. *22*, 101147. https://doi.org/10.1016/j.redox.2019.101147.

Voss, K., Hong, H.S., Bader, J.E., Sugiura, A., Lyssiotis, C.A., and Rathmell, J.C. (2021). A guide to interrogating immunometabolism. Nat. Rev. Immunol. https://doi.org/10.1038/s41577-021-00529-8.

Wynn, T.A., Chawla, A., and Pollard, J.W. (2013). Macrophage biology in development, homeostasis and disease. Nature *496*, 445–455. https://doi.org/10.1038/nature12034.

CellPress
OPEN ACCESS

Yu, W., Wang, X., Zhao, J., Liu, R., Liu, J., Wang, Z., Peng, J., Wu, H., Zhang, X., Long, Z., et al. (2020). Stat2-Drp1 mediated mitochondrial mass increase is necessary for pro-inflammatory differentiation of macrophages. Redox Biol. *37*, 101761. https://doi.org/10.1016/j.redox.2020.101761.

Zhang, I.W., Curto, A., Lopez-Vicario, C., Casulleras, M., Duran-Guell, M., Flores-Costa, R., Colsch, B., Aguilar, F., Aransay, A.M., Lozano, J.J., et al. (2021). Mitochondrial dysfunction governs immunometabolism in leukocytes of patients with acute-on-chronic liver failure. J. Hepatol. https://doi.org/10.1016/j.jhep.2021.08.009.

Zorova, L.D., Popkov, V.A., Plotnikov, E.Y., Silachev, D.N., Pevzner, I.B., Jankauskas, S.S., Babenko, V.A., Zorov, S.D., Balakireva, A.V., Juhaszova, M., et al. (2018). Mitochondrial membrane potential. Anal. Biochem. *552*, 50–59. https://doi.org/10.1016/j.ab.2017.07.009.

STAR★METHODS

KEY RESOURCES TABLE

REAGENT or RESOURCE	SOURCE	IDENTIFIER
Antibodies		
Antibodies and dyes for flow cytometry	This paper (Table S2)	N/A
CD16/CD32 Monoclonal Antibody, Clone 93	eBioScience	Cat#14-0161-86
Human BD Fc Block	BD Biosciences	Cat#564220
Chemicals, peptides, and recombinant proteins		
Lipopolysaccharides from *Escherichia coli* O55:B5	Sigma Aldrich	Cat#L2637
Recombinant Murine IFN-γ	Peprotech	Cat#315-05
Recombinant Human IFN-γ	Peprotech	Cat# 300-02
Recombinant Murine IL-4	Peprotech	Cat#214-14
Recombinant Human IL-4	Peprotech	Cat# 300-02
Lymphoprep^{TM}	Stemcell technologies	Cat#07861
Percoll^{TM}	Cytiva	Cat#17-0891-01
Human M-CSF, research grade	Miltenyi	Cat#130-096-491
Phosphoric acid, 85 wt. %	Sigma Aldrich	Cat#345245
Sulfanilamide	Sigma Aldrich	Cat#S9251
N-(1-Naphtyl)ehtylene diamine dihydrochloride	Sigma Aldrich	Cat#N9125
Triton X-100	Sigma Aldrich	Cat#X100
Tris(hydroxymethyl)aminomethane	Sigma Aldrich	Cat#1083821000
cOmplete^{TM}, Mini Protease Inhibitor Cocktail	Roche	Cat#11836153001
Manganese(II) chloride tetrahydrate	Sigma Aldrich	Cat#M5005
L-arginine	Fluka	Cat#11010
Sulfuric acid, 95.0-98.0%	Fisher Scientific	Cat#15642780
α-isonitrosopropriophenone	Sigma Aldrich	Cat#I3502
Glucose GOD-PAP	Biolabo	Cat#LP80209
meta-Phosphoric acid	Sigma Aldrich	Cat#239275
Glycine	Sigma Aldrich	Cat#15527
Hydrazine hydrate	Sigma Aldrich	Cat#225819
NAD+ (free acid)	Cayman Chemicals	Cat#16077
L-Lactate Dehydrogenase (L-LDH)	Sigma Aldrich	Cat#10127876001
Agilent Seahorse XF Base Medium	Agilent	Cat#102353-100
D-(+)-Glucose solution (45%)	Sigma Aldrich	Cat#G8769
Oligomycin	Sigma Aldrich	Cat#O4876
Carbonyl cyanide 4-(trifluoromethoxy)phenylhydrazone	Sigma Aldrich	Cat#C2920
Antimycin A	Sigma Aldrich	Cat#A8674
Rotenone	Sigma Aldrich	Cat#R8875
Hoechst 33342	Thermo Fisher	Cat#H3570
Saponin	Sigma Aldrich	Cat#SAE0073
Lipofermata	Cayman Chemicals	Cat#25869
Phloretin	Sigma Aldrich	Cat#P7912
Critical commercial assays		
SCENITH kit	Arguello et al. (2020)	http://www.scenith.com
Foxp3 / Transcription Factor Staining Buffer Set	eBioScience	Cat#00-5523-00
PM-M01 kit	Biolog	Cat#13101
Mito-S1 kit	Biolog	Cat#14105

(*Continued on next page*)

Continued		
REAGENT or RESOURCE	SOURCE	IDENTIFIER
Experimental models: Cell lines		
LCM derived from L929 Cell Line from mouse	Sigma Aldrich	L929 cells: Cat#85011425-1VL
Experimental models: Organisms/strains		
C57Bl/6J	Charles River	Strain #632
Buffy coats	Sanquin	Cat# E2824R00
Software and algorithms		
Gen 5^{TM}	BioTek	https://www.biotek.com/products/software-robotics-software/gen5-microplate-reader-and-imager-software/
Wave version 2.6.0.31	Agilent	https://www.agilent.com/en/product/cell-analysis/real-time-cell-metabolic-analysis/xf-software/seahorse-wave-desktop-software-740897
Flow Jo v10	TreeStar	https://www.flowjo.com
OMIQ	OMIQ	https://omiq.ai/
tSNE-CUDA	Chan et al. (2019)	https://github.com/CannyLab/tsne-cuda
Biolog Data Analysis Software version 1.7.1.58	Biolog	https://www.biolog.com/
GraphPad Prism software version 8.2.1	GraphPad Software	https://www.graphpad.com
Other		
XF-96-cell culture plates and cartridges	Agilent	Cat#101085-004
RPMI 1640 Medium, no glucose	Gibco	Cat#11879020

RESOURCE AVAILABILITY

Lead contact

Further information and requests for resources and reagents should be directed to and will be fulfilled by the lead contact, Jan Van den Bossche (j.vandenbossche@amsterdamumc.nl).

Materials availability

This study did not generate new unique reagents. As described previously, adapted SCENITH protocols and all the reagents including the panel of inhibitors, puromycin and the monoclonal antibody clone R4743L-E8, conjugated with Alexa Fluor 647 or Alexa Fluor 488 (SCENITH kit) are available upon application at http://www.scenith.com/.

Data and code availability

All data reported in this paper will be shared by the lead contact upon request. This paper does not report original code. Any additional information required to reanalyze the data reported in this paper is available from the lead contact upon request.

EXPERIMENTAL MODEL AND SUBJECT DETAILS

Bone marrow isolation and BMDM culture

Mouse experiments were approved by the Committee for Animal Welfare of the VU University Amsterdam. 8–16 week old male and female C57Bl/6J mice were purchased from Charles River and housed in groups of four in SPF conditions at 21°C until sacrifice. Bone marrow cells were isolated from femurs and tibias by flushing with PBS. Bone marrow-derived macrophages (BMDMs) were generated by culturing in 145 × 20 mm petri dishes (greiner bio-one) in 20 ml complete RPMI-1640 (Gibco) containing 2 mM L-glutamine, 10% FCS (Gibco), 100 U/ml penicillin, 100 μg/ml streptomycin (all Gibco), and 15% L929-conditioned medium (LCM) for 6 days, which resulted in >90% macrophage purity (Figure S1B). 10 ml fresh medium was added on day 3. On day 6, cells were harvested with cold PBS and gentle scraping and counted using a Bürker cell counting chamber with 0.0025 mm^2 grid (Optik Labor). Cells were subsequently plated at a density of $1*10^6$ cells/ml in fresh medium with 5% LCM in 96-well plates for the experiments. On day 7, medium was refreshed and cells were either left untreated or stimulated with 100 ng/ml LPS (Sigma Aldrich), 10 ng/ml LPS+100 U IFNγ (Peptrotech), or 20 ng/ml IL-4 (Peprotech) for 24 hours in the presence of 5% LCM.

Monocyte isolation and HMDM culture

Buffy coats (50 ml) were purchased from Sanquin blood Bank (Amsterdam, Netherlands). Information regarding sex and age of donors was not supplied by the blood bank. PBMCs were isolated with a Ficoll/Lymphoprep gradient (Stemcell technologies) and

careful centrifugation at 800g for 30 minutes. Next, monocytes were isolated by applying 120-150*10^6 cells on top of a 46% Percoll™ (Cytiva) solution followed by careful centrifugation at 2000 rpm for 20 minutes. Monocytes were counted in the same manner as BMDMs and plated at a density of 2*10^6 cells/ml in the appropriate 96-well plates for each experiment in 100 μl IMDM medium containing HEPES (Gibco) supplemented with 2 mM L-glutamine, 100 U/ml penicillin, 100 μg/ml streptomycin (full IMDM medium), and containing 1% FCS. After settling for one hour, medium was replaced with full IMDM medium with 10% FCS and 50 ng/ml M-CSF (Miltenyi) for 6-day differentiation, resulting in >95% macrophage purity (Figure S1C). On day 3, medium was replaced with fresh medium supplemented with M-CSF. On day 6, cells (near 100% macrophage purity (Figures S1A and S1C)) were left untreated or stimulated in fresh medium without M-CSF with 100 ng/ml LPS, 10 ng/ml LPS + 20 ng/ml IFNγ (Peprotech), or 20 ng/ml IL-4 (Peprotech) for 24 hours.

METHOD DETAILS

NO production and arginase activity assay

100 μl supernatant was collected from 96-well plates with 1*10^5 cells (BMDMs/HMDMs) per well. Subsequently, NO production was measured by adding 50 μl Griess reagent (2.5% H_3PO_4 (Merck), 1% sulfanilamide (Sigma Aldrich), and 0.1% naphtylene diamide dihydrochloride (Sigma Aldrich) in H_2O) to 50 μl cell supernatants (1:1) and optical density was measured at 540 nm.

Arginase activity was determined on cell lysates of 5*10^4 cells (BMDMs) or 1*10^5 cells (HMDMs). Cells were washed with PBS and lysed by incubating for 30 minutes with 100 μl 0.1% Triton X-100 (Sigma Aldrich), 25 mM Tris-HCl (pH 7.5, Sigma Aldrich) supplemented with 1x protease inhibitor cocktail (Roche). Arginase was activated by adding 3.5 μl of 10 mM $MnCl_2$ (Sigma Aldrich) to 10 μl sample and incubated at 56°C for 10 minutes. Next, samples were incubated with 10 μl 0.5 M L-arginine (pH 9.7, Fluka) for 60 minutes at 37°C. The reaction was stopped by adding 90 μl stop solution (96%H_2SO_4/85% H_3PO_4/H_2O 1:3:7, Merck) and incubated with 4 μl α-isonitrosopropiophenone (9%, Sigma Aldrich) for 30 minutes at 95°C. Samples were left in the dark to cool down to room temperature until measurement of optical density at 540 nm. Enzymatic activity was calculated by [Urea]*(total volume*10^6)/(tested volume*Time(incubated at 37°C)*1000).

Glucose consumption assay

100 μl supernatant was collected from 96-well plates with 1*10^5 cells (BMDMs/HMDMs) per well. To determine glucose levels in supernatant, samples and standard (5 μl per well) were pipetted into a 96-well plate. 250 μl glucose reagent (BIOLABO) was added to standard and samples, mixed by pipetting and incubated for 30 minutes in the dark. Absorbance was measured at 490 nm. Glucose consumption was calculated as the difference between glucose levels of medium without cells and glucose levels in cell supernatants.

Lactate production assay

100 μl supernatant was collected from 96-well plates with 1*10^5 cells (BMDMs/HMDMs) per well. Lactate levels in cellular supernatants were determined by conversion of lactate into NADH by lactate dehydrogenase (LDH). First, samples were incubated for 15 minutes at 4°C with 3% metaphosphoric acid (Sigma Aldrich), centrifuged at 20.000 g for 10 minutes, and supernatants were used further. 5 μl of thus deproteinized samples were transferred into a 96-wells plate and 150 μl Master Mix consisting of 0.5 M Glycine - 0.4 M hydrazine buffer (pH = 9.0, Sigma Aldrich) with 27 mM NAD (Cayman Chemicals) was added per well. NADH fluorescence was measured using a Mithras LB 940 with $\lambda_{ex}/\lambda_{em}$= 340-10 /450-10 nm every 2 minutes for 5 cycles as background measurement. Next, 50 μl of start solution consisting of 0.5 M Glycine - 0.4 M hydrazine buffer (pH = 9.0) and 5 mg/ml LDH (Sigma Aldrich) was added to each well. Fluorescence was measured every 2 minutes with shaking until a stable read was achieved.

Extracellular flux analysis

XF analysis was performed using the Seahorse XFe-96 Flux Analyzer (Agilent) to examine oxygen consumption (OCR) and extracellular acidification rates (ECAR) as described previously (Van den Bossche et al., 2015). Briefly, BMDMs and HMDMs were plated at a density of 7.5*10^4 cells per well in XF-96-cell culture plates (Agilent) and stimulated for 24 hours with LPS or IL-4, or left untreated. 1 hour prior to the assay, cells were washed and medium was replaced by Seahorse base medium (Agilent) without glucose, phenol red, and sodium bicarbonate, supplemented with 5 mM HEPES and 2 mM L-glutamine. The run consisted of 2 minutes mixing, 3 minutes measuring and subsequent 4 injections; Glucose (final concentration in well 25 mM), Oligomycin (O, final concentration 1.5 μM), FCCP (final concentration 1.5 μM), and antimycin A (AA, final concentration 2.5 μM) with rotenone (rot, final concentration 1.25 μM, all Sigma Aldrich) and Hoechst 33342 (Thermo Fisher) (final concentration 5 μg/ml). Directly after the run, Hoechst signal was measured on the Cytation 5 Cell Imaging multi-mode reader (BioTek) with a 4X magnification using a 365 nm LED in combination with an EX377/50 EM 447/60 filter cube and cell counts were analyzed using Gen 5™ software. Subsequently, flux rate data was normalized to cell counts with the following equation:

$$\textit{Normalized OCR or ECAR} = \textit{OCR or ECAR} \Big/ \frac{\textit{cell count in center of well}}{\textit{average of plate}}.$$

Data were analyzed using Wave software version 2.6.0.31 as described previously (Van den Bossche et al., 2015).

Mitochondrial and glycolytic contributions to total ATP production rate were calculated as described here (Romero et al., 2017). First, OXPHOS-related acidification (mitochondrial proton efflux rate, mitoPER) of the assay medium was calculated as follows:

$$mitoPER = CCF * \left(OCR_{basal} - OCR_{Rot/AA}\right)),$$

where CCF (CO_2 contribution factor) was determined as 0.61 for XFe96 plates by the manufacturer for a range of cell types. Total proton efflux rate (PER) can be calculated as:

$$total\ PER = ECAR * BF * Vol_{measurement\ chamber} * K_{vol},$$

where the buffer factor (BF) of the medium refers to the amount of H^+ necessary to change the pH of the medium by one unit, and was measured according to manufacturer instructions in the Buffer Factor Protocol Quick Reference Guide (Agilent). $Vol_{measurement\ chamber}$ and K_{vol} are scaling factors to determine the effective volume of the well and were determined as 2.28 μl and 1.60, respectively, by the manufacturer for XFe96 plates. From this, glycolytic proton efflux rate and therefore glycolytic ATP production rate can be determined:

$$glycoATP = glycoPER = total\ PER - mitoPER.$$

Next, mitochondrial ATP production rate can be calculated as follows:

$$mitoATP = \left(OCR_{basal} - OCR_{Rot/AA}\right) * 2 * P/O,$$

where multiplication by 2 is a stoichiometric correction for oxygen atoms consumed, and P/O is the number of ADP molecules phosphorylated to ATP per atom of oxygen which was determined by the manufacturer as 2.75 for a range of cell types. Lastly, total ATP production rate is the sum of glycoATP and mitoATP, as described by the manufacturer in the Real-time ATP Rate assay Kit (Agilent).

SCENITH and flow cytometry and analysis

SCENITH protocol was performed as described previously (Arguello et al., 2020). Briefly, control or metabolic inhibitors Deoxy-D-glucose (DG, final concentration 100 mM), oligomycin (O, final concentration 1 μM), combination of DG and O (DGO), or Harringtonine (H, final concentration 2 mg/ml) as negative control were added to fully differentiated cells and incubated for 15 min at 37°C. Subsequently, puromycin (final concentration 10 μg/ml) was added without washing and incubated for another 30 min at 37°C. After incubation, cells were washed with cold PBS, harvested by incubating with PBS + 5 mM EDTA for 10 min at 4°C and proceeded with Fc receptor blockade (eBioScience for mouse, BD Biosciences for human cells) and fixable viability dye (eBioScience) staining for 15 min at 4°C in the dark. Subsequently, cells were washed and stained with surface antibody mix in PBS/0.5% BSA/0.02% sodium azide (PBA) for 30 min at 4°C in the dark. Cells were then washed, fixed and permeabilized using the FOXP3 fixation and permeabilization kit (eBioScience) according to manufacturer's instructions. For intracellular staining of iNOS, Arg1 and puromycin, cells were incubated for 1h at 4°C in antibody staining solution in permeabilization buffer. Samples were then transferred to a plate-reader compatible 96-well U-bottom plate.

All samples were acquired within 24 hours of the experiment at the O2 Flow Facility at Amsterdam UMC (Netherlands) on an X20 Fortessa flow cytometer (BD Biosciences) with high-throughput sampler. The flow cytometer was calibrated daily using CS&T calibration beads (BD Biosciences).

Data were analyzed using FlowJo (TreeStar, v10) and were compensated using single stains with UltraComp eBeads (ThermoFisher) labeled with the appropriate fluorochrome. Next, cells were gated on FSC-A/SSC-A to gate out debris, then on FSC-A/FSC-H to identify single-cells and lastly on FVD^- viable cells (Figure S1A).

For unbiased tSNE analyses, files of oligomycin-treated cells were uploaded to the OMIQ online analysis platform (https://omiq.ai/), scaled and subsampled to include 10.000 cells live single cells per file. Next, the tSNE-CUDA (Chan et al., 2019) tool set to 1500 iterations, a perplexity of 30 and a theta of 0.5 was used to create tSNE dimensionality reduction. Cells were overlaid on tSNE dimensionality reduction according to stimulus. Stimulus-associated 'clusters' were further assessed using the 'Clustered Heatmap' tool in OMIQ to identify discriminating markers between activation states.

Vehicle control, DG, O, Harringtonine, puromycin and anti-puromycin antibodies were received as SCENITH kit from (http://www.scenith.com) (Arguello et al., 2020). A complete list of antibodies used can be found in Table S2.

Fluorescent metabolic dyes

Mitochondrial mass was measured using MitoTracker Green (Invitrogen). Mitochondrial membrane potential was measured using Tetramethylrhodamine methyl ester (TMRM, Thermo Fisher) and fatty acid and glucose uptake was measured using BODIPY™ FL C16 (BODIPY, Thermo Fisher) or 2-(N-(7-Nitrobenz-2-oxa-1,3-diazol-4-yl)Amino)-2-Deoxyglucose (2NB-DG, Invitrogen) fluorescent probes. Cells were plated in 96-well black culture plates at a density of $8*10^4$ cells/well. Cells were starved for 2 hours in basal RPMI-medium with (for BODIPY C16) or without glucose (for 2NB-DG) and subsequently stained by incubation for 30 minutes in complete RPMI with either 100 nM MitoTracker, 100 nM TMRM, 100 μM 2NB-DG or with 0.75 μM BODIPY. Concentrations were determined by analysis of unspecific staining and effect on cell viability (Figure 4). Hoechst (final concentration 5 μg/ml) was added for the last 5 minutes of incubation. Cells were washed with PBS

and imaged in 4X magnification on the Cytation 5 at 37°C using a 465 nm LED in combination with an EX 469/35 EM 525/39 filter cube for MitoTracker Green, BODIPY and 2NB-DG and a 523 nm LED in combination with an EX 531/40 EM 593/40 filter cube for TMRM.

For FACS analysis, cells were harvested using ice-cold PBS+5 mM EDTA, transferred to a plate-reader compatible 96-well U-bottom plate and immediately acquired on an X20 Fortessa flow cytometer with plate reader (BD Biosciences) and analyzed as described in the section 'Flow cytometry and analysis'. To validate metabolic dye signal, MitoTracker Green and TMRM signal were inhibited by the uncoupler FCCP (Sigma-Aldrich, 5 μM final concentration), 2NB-DG by phloretin, an inhibitor of glucose transport (Sigma-Aldrich, 150 μM final concentration), and BODIPY C16 by lipofermata, an inhibitor of fatty acid transporter 2 (Cayman Chemicals, 10 μM final concentration) (Figure 4).

Mitochondrial functional substrate assay

Mitochondrial functional substrate assays were performed using mitochondrial-substrate-coated plates (Biolog). First, cells for mitochondrial functional substrate assays were seeded in a 96 well culture plate at a density of $8*10^4$ cells/well and stimulated for 24 hours with LPS or IL-4, or left untreated. 1 hour prior to the assay, 30 μl assay buffer with 1X saponin (Sigma-Aldrich, final concentration 50 μg/ml) and redox dye MC was dispensed into the wells of the mitochondrial-substrate-coated plate and incubated at 37°C. Then, cells were washed once with 100 μl assay buffer and subsequently incubated with 35 μl assay buffer with 1X saponin at room temperature for permeabilization. After 15 minute incubation time, permeabilized cells were mixed by pipetting up and down and incubated for another 15 minutes at room temperature. Lastly, 30 μl of permeabilized cell suspension in 1X saponin was transferred to the mitochondrial-substrate-coated plate.

Additionally, intact BMDMs were plated as $4*10^4$ cells/well in 50 μl MC-0 Assay Medium into carbon-substrate-coated plates (Biolog) to further assess whole-cell substrate usage as opposed to only mitochondrial substrate use. Cells and substrates were pre-incubated for 24 hours at 37°C and 5% CO_2, and metabolism was assessed after adding 20 μl 6X Biolog Redox Dye MB.

Color formation in mitochondrial-substrate-coated and carbon-substrate-coated plates was measured by the automated platform OmniLog (Biolog) and maximum rates between 1 and 4 hours of analysis were determined using Biolog Data Analysis software version 1.7.1.58.

QUANTIFICATION AND STATISTICAL ANALYSIS

Data are presented as mean ± standard error of the mean (SEM) unless specified differently. Statistical significance was analyzed using an ordinary one-way or two-way ANOVA where appropriate followed by Sidak's or Dunnett's correction for multiple comparisons, respectively, in GraphPad Prism software (8.2.1) using paired analysis to minimize effects of mouse/donor variation. For substrate utilization experiments, significant substrates were determined with a Student's t-distribution using Biolog Data Analysis Software. p values <0.05 were considered statistically significant indicated by $*p < 0.05$, $**p < 0.01$, $***p < 0.001$. The number of mice or donors included in each experiment is indicated in the figure legend. Except for the titration of metabolic dyes, all experiments were performed as at least 2 independent experiments. For NO and Arginase assays, XF analyses and fluorescent probe uptake, at least 3 technical replicates were included to calculate a mean value to represent a mouse or human donor.

Cell Reports Methods, Volume *2*

Supplemental information

An integrated toolbox to profile macrophage immunometabolism

Sanne G.S. Verberk, Kyra E. de Goede, Friederike S. Gorki, Xanthe A.M.H. van Dierendonck, Rafael J. Argüello, and Jan Van den Bossche

Supplementary information

Supplementary Table 1: Required optimizations for techniques, related to Table 1.

Technique	Required optimization
**Arginase assay**	Seeding density, duration of enzymatic reaction
**XF analyzer**	Seeding density, coating of plates, FCCP concentration
**SCENITH**	Timing of incubation with inhibitors and puromycin, stability of other activation or metabolic markers in flow cytometry upon treatment with inhibitors
**Metabolic dyes**	Optimal concentration and timing of dye: specificity of staining, viability, optimal fluorescent intensity; MitoTracker Green: independence of mitochondrial membrane potential

Supplementary Table 2: List of antibodies used, related to STAR Methods

Target	Species	Clone	Fluorochrome	Company	Identifier
**CD40**	M	3/23	Pacific Blue	BioLegend	Cat#124625
**CD80**	M	16-10A1	BV650	BioLegend	Cat#104732
**CD86**	M	GL-1	BV510	BioLegend	Cat#105040
**iNOS**	M	CXNFT	APC	eBioScience	Cat#17-5920-80
**CD206**	M	C068C2	BV605	BioLegend	Cat#141721
**CD301b**	M	URA-1	PE-Cy7	BioLegend	Cat#146808
**CD273**	M	TY25	PE-CF594	BioLegend	Cat#107215
**Arg1**	M	A1exF5	PE	eBioScience	Cat#17-3697-80
**F4/80**	M	BM8	BV711	BioLegend	Cat#123147
**CD11b**	M/H	M1/70	PE-Cy7	BioLegend	Cat#101216
**CD40**	H	5C3	BV785	BioLegend	Cat#334340
**CD197**	H	G043H7	BV421	BioLegend	Cat#353207
**CD80**	H	2D10	PE-Cy7	BioLegend	Cat#305218
**CD273**	H	MIH18	PE	BioLegend	Cat#345505
**CD200R**	H	OX-108	PE-CF594	BioLegend	Cat#329310
**CD206**	H	15-2	APC	BioLegend	Cat#321110
**HLA-DR**	H	G46-6	BV510	BD Biosciences	Cat#563083
**CD14**	H	63D3	AF700	BioLegend	Cat#367114
**CD68**	H	Y1/82A	PE	BioLegend	Cat#333808
**Fixable Viability Dye**	n/a	n/a	eFluor780	eBioScience	Cat#65-0865-14
**Puromycin**	M/H	R4743L-E8	AF488	SCENITH kit http://www.scenith.com/ (Arguello et al, 2020)	n/a
**MitoTracker Green**	n/a	n/a	490/516 nm	ThermoFisher	Cat#M7514
**TMRM**	n/a	n/a	548/574 nm	ThermoFisher	Cat#T668
**2NB-DG**	n/a	n/a	~465/540 nm	Invitrogen	Cat#N13195
**BODIPY C16**	n/a	n/a	505/512 nm	ThermoFisher	Cat#D3821

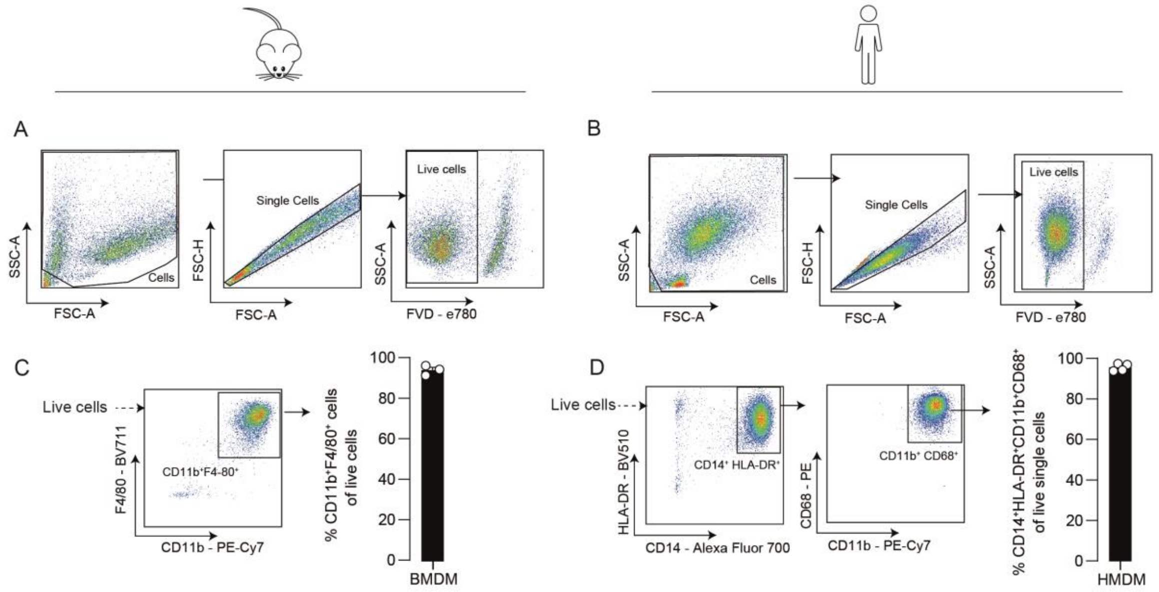

Supplementary Figure 1: Culturing of BMDMs and HMDMs leads to pure macrophage populations, related to Figures 2-5. (A, B) Gating strategy for live single cells of BMDMs (A) and HMDMs (B). (C) Gating and quantification of $CD11b^+F4/80^+$ BMDMs. (D) Gating and quantification of $CD14^+HLA\text{-}DR^+CD11b^+CD68^+$ HMDMs. Gates were set according to FMO controls. N=3 mice or N=4 donors with 4 technical replicates in 2 independent experiments.

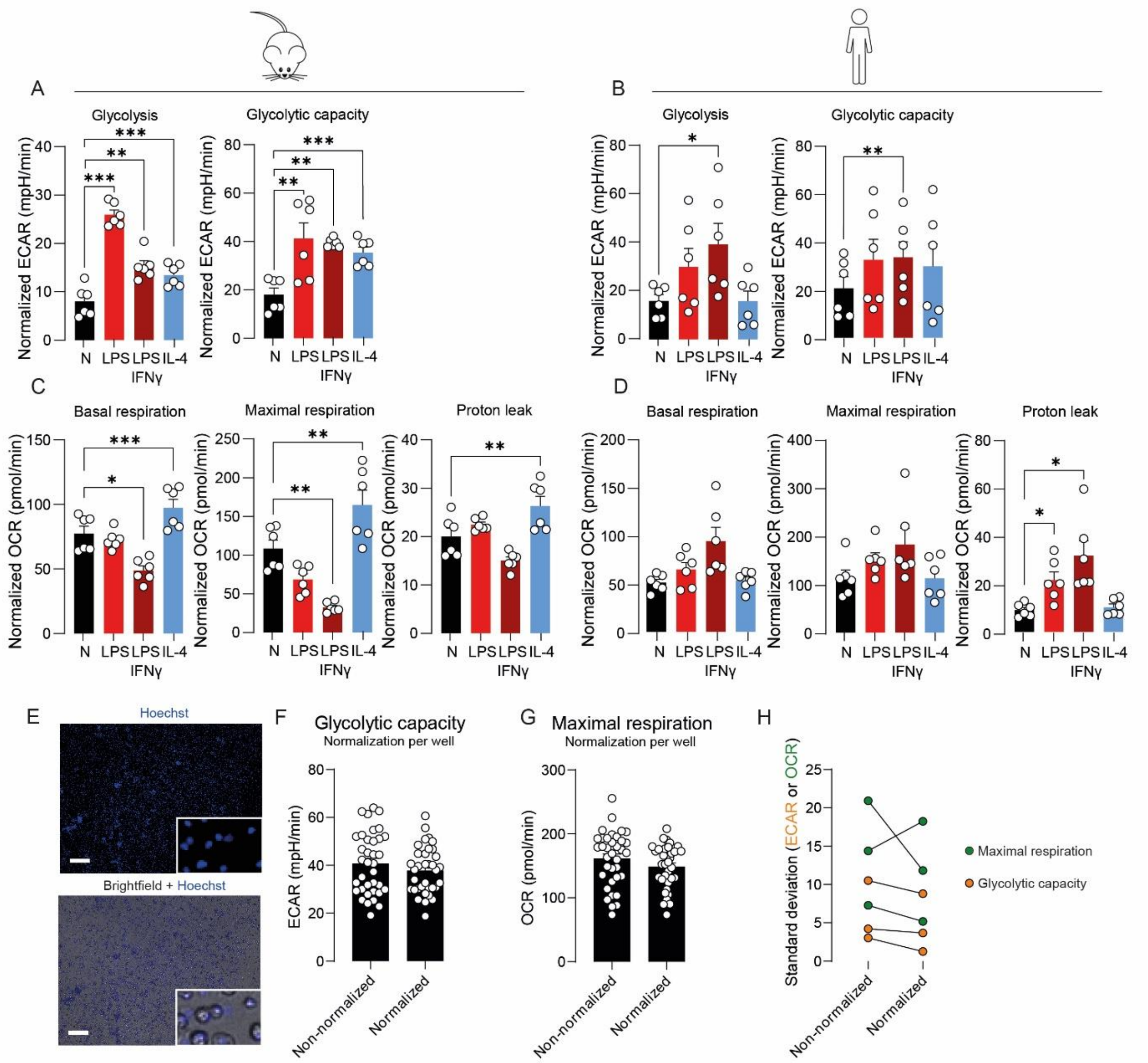

Supplementary Figure 2: Additional XF-derived parameters and normalization for cell counts after cell permeable Hoechst staining, related to Figure 3. (A, B) XF parameters extracted from ECAR data in BMDMs (A) and HMDMs (B) stimulated with LPS±IFNγ, IL-4 or left untreated. Data is normalized to relative Hoechst^{+} objects per experiment. (C, D) XF parameters extracted from ECAR data in BMDMs (C) and HMDMs (D) stimulated with LPS±IFNγ, IL-4 or left untreated. Data is normalized to relative Hoechst^{+} objects per experiment. (E) Hoechst staining of cells in Seahorse plate for normalization, captured in fluorescent mode and overlaid with brightfield picture to validate recognition of cells. Scale bar represents 200 µm. (F, G) Glycolytic capacity (F) and maximal respiration (G) of all measured wells before and after normalization with relative cell counts determined by Hoechst staining in 3 different experiments, each exploring unstimulated BMDMs from 3 different mice. (H) Standard deviations of XF parameters derived from combining data of 3 mice within 1 experiment, before and after normalization for relative cell count (N=3 experiments). Data are shown as mean ± SEM (A-D, F, G), individual data points indicate a mouse (N=6) (A, C), human donor (N=6) (B, D) or a separate well (F, G). Alternatively, individual data points indicate standard deviation in 1 experiment (H). * $P<0.05$, ** $P<0.01$, *** $P<0.001$ by one-way ANOVA with Dunnett's post-hoc test for multiple comparisons.

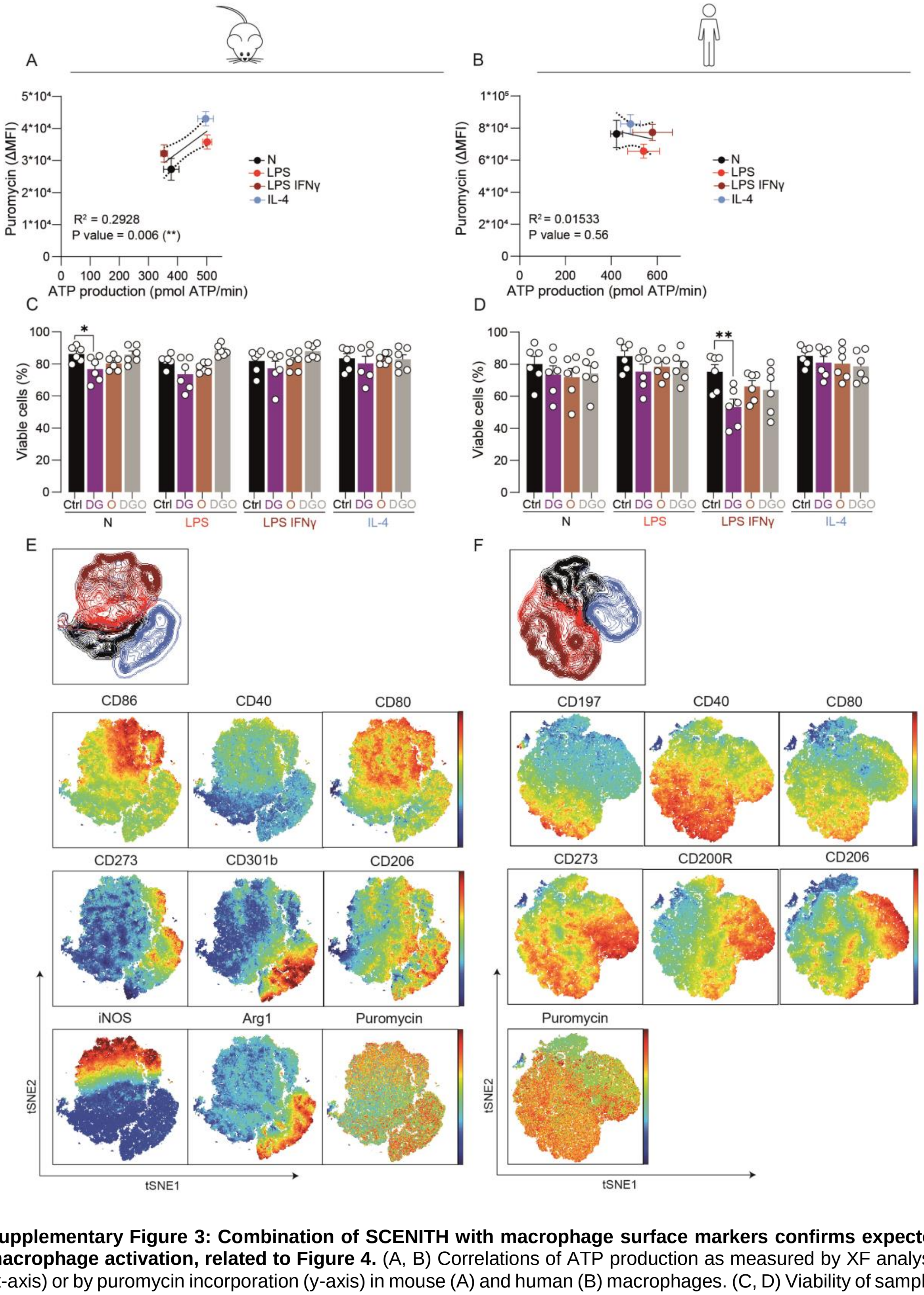

Supplementary Figure 3: Combination of SCENITH with macrophage surface markers confirms expected macrophage activation, related to Figure 4. (A, B) Correlations of ATP production as measured by XF analysis (x-axis) or by puromycin incorporation (y-axis) in mouse (A) and human (B) macrophages. (C, D) Viability of samples treated with different metabolic inhibitors during SCENITH protocol for comparison of naïve, LPS±IFNγ- and IL-4-activated BMDMs (C) and HMDMs (D) as determined by fixable viability dye. (E, F) tSNE dimensionality reduction and marker expression of oligomycin-treated BMDMs (E) and HMDMs (F). Data are shown as mean ± SEM. In C and D, each dot marks a separate mouse (N=6) or donor (N=6). * P<0.05, ** P<0.01 by two-way ANOVA with Dunnett's post-hoc test for multiple comparisons. Correlations were fitted using a simple linear regression model (A, B).

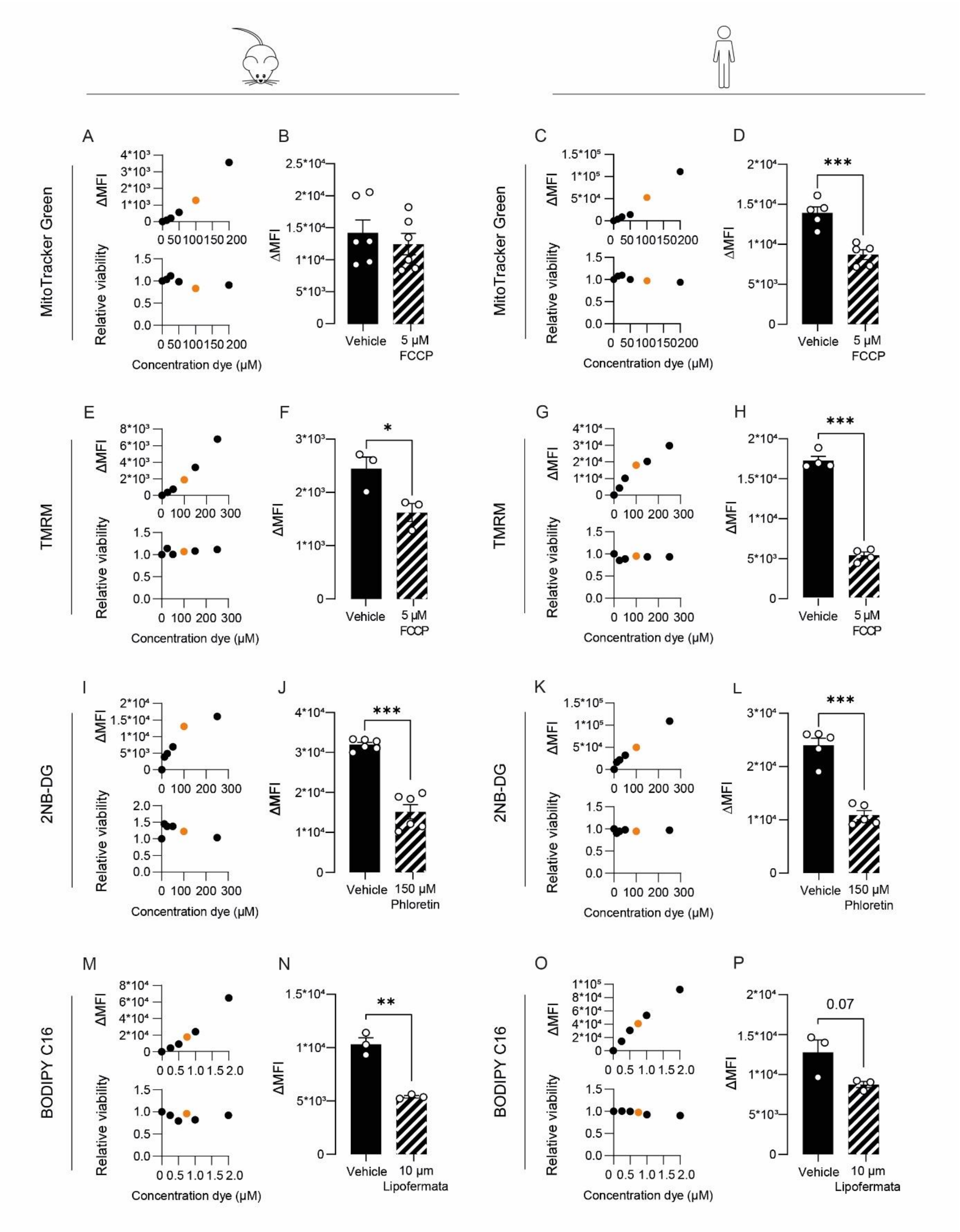

Supplementary Figure 4: Validation of metabolic dye concentration and signal measured by flow cytometry, related to Figure 5. Dose response curves of fluorescent signal and viability measured by flow cytometry or Cytation as a result of increasing metabolic dye concentration, and decrease of fluorescent signal upon inhibition for MitoTracker Green (A-D), TMRM (E-H), 2NB-DG (I-L) and BODIPY C16 (M-P) in BMDMs (left) and HMDMs (right). Orange dots indicate the concentration of the dye chosen for further experiments. Data are shown as mean from 2-3 technical replicates (A, C, E, G, I, K, M, O) or mean ± SEM where each dot marks the average of a technical duplicate for a separate mouse (N=3-6) or donor (N=3-6)(B, D, F, H, J, L, N, P). ΔMFI was calculated as MFI (median fluorescent intensity) of sample – MFI of unstained control. * $P<0.05$, ** $P<0.01$, *** $P<0.001$ by unpaired two-tailed *t*-test.

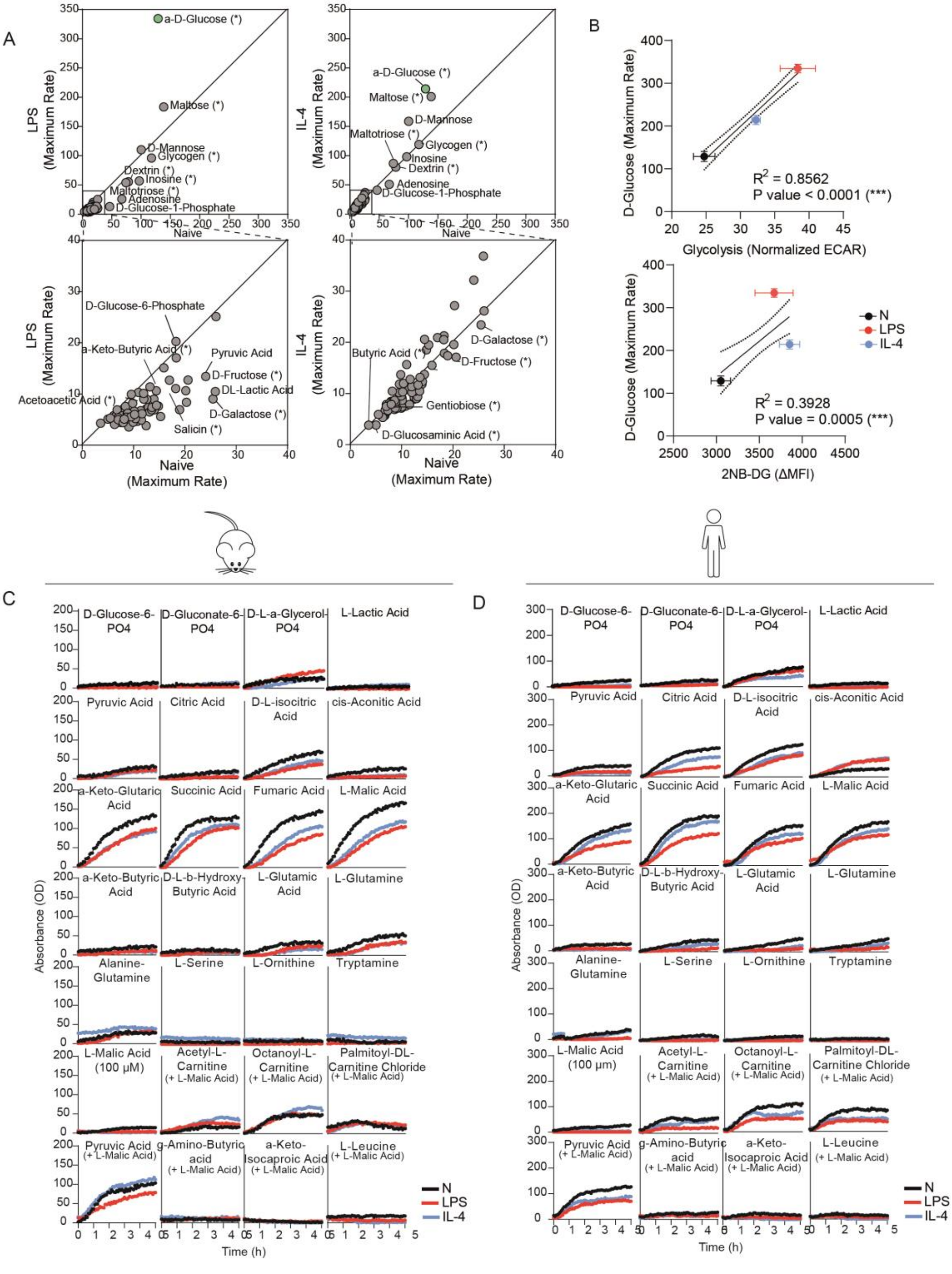

Supplementary figure 5: Assessment of substrate oxidation provides insight in glucose use and specific mitochondrial enzymatic activity by activated macrophages, related to STAR methods (Mitochondrial functional substrate assay). (A) Scatterplot comparisons of maximum rate in the timespan of 1-4h of substrate utilization by intact LPS- and IL-4-activated versus naive BMDMs. (B) Correlation of maximum D-glucose uptake as measured by carbon-substrate-coated plates and glycolysis as measured by XF analysis and 2NB-DG as measured by flow cytometry. (C,D) Kinetic rates of substrate usage by permeabilized BMDMs (C) and HMDMs (D). Correlations were fitted using a simple linear regression model (B).

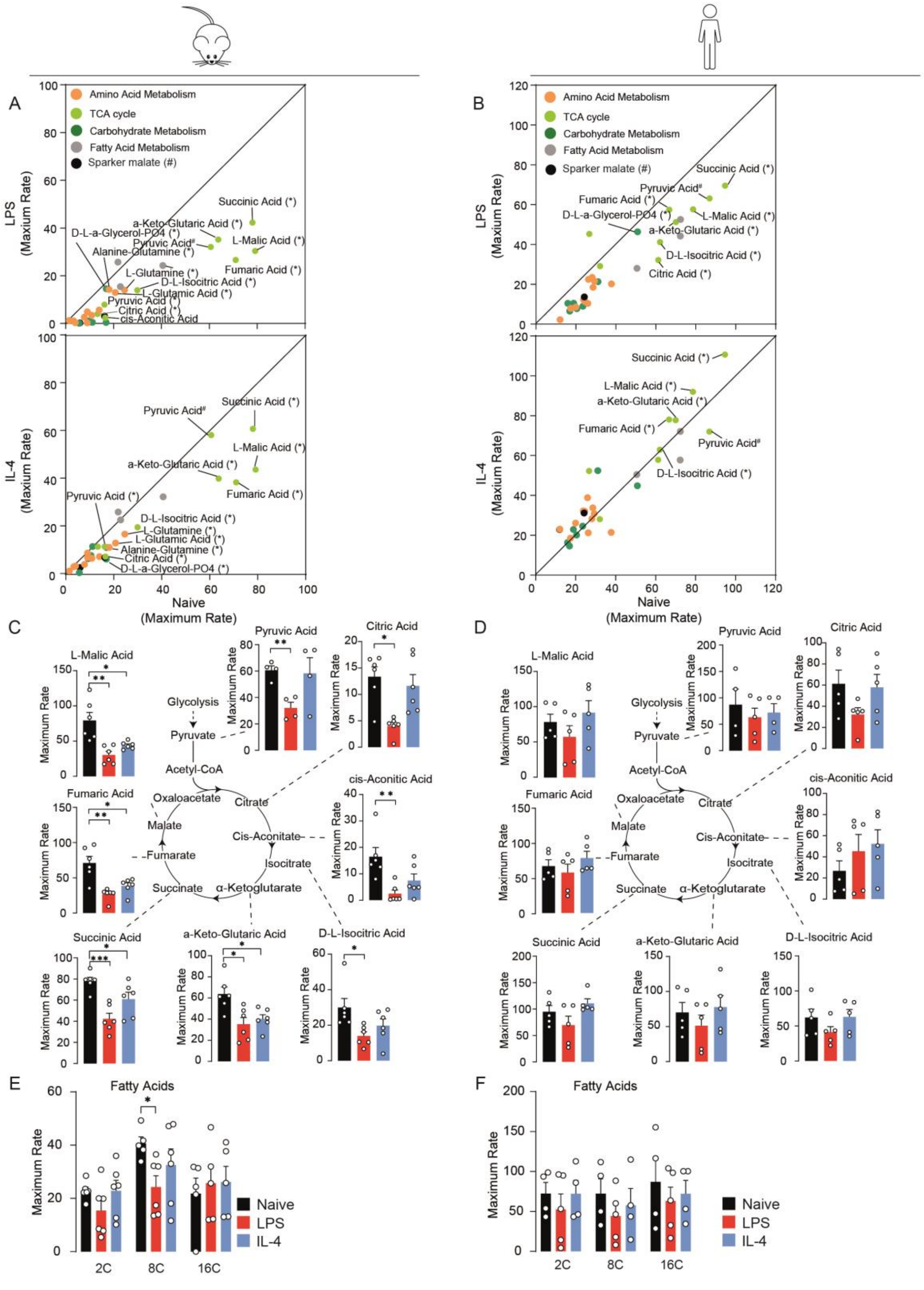

Supplementary Figure 6: Assessment of mitochondrial substrate oxidation by permeabilized cells provides insight in TCA cycle substrate and fatty acid oxidation, related to STAR methods (Mitochondrial functional substrate assay). (A, B) Scatterplot of maximum rate in the timespan of 1-4h of substrate oxidation by LPS- and IL-4-activated compared to naïve permeabilized BMDMs (A) and HMDMs (B). Substrates belonging to amino acid metabolism, TCA cycle, glycolysis and fatty acid metabolism are depicted in yellow, light green, dark green and grey, respectively. Significantly different substrates are marked with (*) and substrates supplemented with sparker malate with $^{\#}$. (C, D) Maximum rate of the oxidation of specific TCA-cycle substrates in permeabilized BMDMs (C) and HMDMs (D). (E, F) Oxidation of short- (2C), medium- (8C), and long-chain fatty acids (16C) in BMDMs (E) and HMDMs (F). Data are shown as mean (maximum) rate for all mice or donors. * P<0.05 by unpaired Student's t-tests (A, B).Data are shown as mean ± SEM. In C-F, each dot marks a separate mouse (N=4-5) or donor (N=4-5). * P<0.05, ** P<0.01, *** P<0.001 by one-way ANOVA with Sidak's post hoc-test for multiple comparisons.
